# NOTCH3 regulates myofibroblastic CAF differentiation via the P62–ROS signaling axis to promote bladder cancer progression

**DOI:** 10.1186/s13046-026-03751-1

**Published:** 2026-06-06

**Authors:** Ding Hu, Chengquan Shen, Changxue Liu, Fei Xie, Xinning Wang, Hexiang Wang, Yunqing Chen, Xinzhao Zhao, Ruize Qin, Cheng Li, Huaixi Ge, Jianhang Zhu, Zhengze Xi, Yonghua Wang

**Affiliations:** 1 Department of Urology, The Affiliated Hospital of Qingdao University, Qingdao, Shandong China; 2 Urinary Diseases Clinical Medical Research Center of Qingdao, Qingdao, Shandong China; 3 Shandong Province Medical and Health Key Laboratory of Urology, Qingdao, Shandong China; 4 Department of Radiology, The Affiliated Hospital of Qingdao University, Qingdao, Shandong China; 5 Department of Pathology, The Affiliated Hospital of Qingdao University, Qingdao, Shandong China

**Keywords:** NOTCH3, Cancer-associated fibroblasts, Bladder cancer, Reactive oxygen species

## Abstract

Cancer-associated fibroblasts (CAFs) are important contributors to malignant progression in bladder cancer (BLCA), yet the mechanisms by which they promote tumor progression remain incompletely understood. In this study, we integrated patient-derived CAFs with bulk and single-cell transcriptomics, spatial transcriptomics, and multi-cohort clinical datasets to systematically define the molecular mechanisms underlying CAF-driven BLCA progression. Integrated analyses of bulk RNA sequencing, single-cell transcriptomics, spatial transcriptomics, immunohistochemistry, and multiplex immunofluorescence of paraffin-embedded tissues, together with PCR, immunoblotting, and immunofluorescence in patient-derived normal fibroblasts and CAFs, consistently demonstrated marked enrichment of NOTCH3 in CAFs in bladder cancer. Silencing of NOTCH3 attenuated extracellular matrix deposition, contractile capacity, intracellular ROS accumulation, and the ability of CAFs to support tumor growth. Mechanistically, TGF-β induced NOTCH3 transcription through SMAD2 binding to the NOTCH3 promoter. In turn, NOTCH3 recruited HSPA8 to promote K48-linked ubiquitination and proteasomal degradation of P62, thereby suppressing the P62–NRF2 antioxidant pathway and sustaining intracellular ROS accumulation. Restoration of P62 expression or pharmacological scavenging of ROS effectively reversed NOTCH3-driven myofibroblastic differentiation and stromal support of tumor growth. In vivo, CAF-specific deletion of NOTCH3 significantly inhibited tumor growth, reduced collagen deposition, and attenuated stromal remodeling in both subcutaneous and orthotopic bladder cancer models. Collectively, these findings identify CAF-intrinsic NOTCH3 as important regulator of myofibroblastic differentiation through integration of upstream TGF-β signaling with redox and proteostatic control, highlighting stromal NOTCH3 as a potential therapeutic target to limit malignant progression in BLCA.

**Supplementary Information:**

The online version contains supplementary material available at 10.1186/s13046-026-03751-1.

## Background

Bladder cancer (BLCA) remains a clinically challenging malignancy with high recurrence rates, limited therapeutic durability, and substantial interpatient heterogeneity [1, 2]. Although immune checkpoint blockade and multimodal therapies have improved outcomes in a subset of patients, overall response rates remain modest and resistance is common [3, 4]. Increasing evidence suggests that these limitations are not solely driven by tumor-intrinsic alterations but are critically shaped by the tumor microenvironment (TME), where cancer-associated fibroblasts (CAFs) play central roles in stromal remodeling, immune suppression, and extracellular matrix deposition [5–7].

Single-cell and spatial transcriptomic studies have revealed marked CAF heterogeneity, identifying functionally distinct subsets including inflammatory CAFs (iCAFs), antigen-presenting CAFs (apCAFs), and myofibroblastic CAFs (myCAFs) [8–14]. Among these, myCAFs are characterized by enhanced contractility and extracellular matrix production and are strongly associated with tumor progression and poor therapeutic response in BLCA [15–18].

TGF-β signaling is a key driver of CAF activation and myCAF differentiation [19–21]. However, the mechanisms that sustain myCAF commitment within the dynamic TME remain incompletely understood. Emerging evidence indicates that myCAF specification is reinforced not only by transcriptional programs but also by redox homeostasis [22, 23]. Persistent accumulation of reactive oxygen species (ROS) acts as a key amplifier of contractility and extracellular matrix remodeling, thereby stabilizing the tumor-promoting myCAF phenotype [20, 22]. Yet, the upstream pathways governing ROS homeostasis during myCAF formation remain largely undefined.

Pathways integrating differentiation signals with stress adaptation may underlie stable myCAF specification. NOTCH signaling, a conserved regulator of cell fate and tissue remodeling, has been implicated in fibrosis and tumor progression [24, 25]. Notably, our previous work has demonstrated a tumor-promoting role of NOTCH3 in bladder cancer, suggesting that NOTCH3 may function as a key mediator linking tumor-intrinsic signaling to microenvironmental regulation [26]. However, its receptor-specific roles within CAF subsets remain unclear.

In this study, we aimed to elucidate the coupling of TGF-β signaling with sustained redox regulation and myCAF commitment in BLCA. Integrating patient-derived CAFs, multi-omics profiling, and functional–mechanistic analyses, we sought to define the signaling axis governing myCAF stability and its contribution to tumor progression.

## Methods

### Isolation and culture of primary CAFs

Primary CAFs were isolated from tumor tissues of 20 patients with pathologically confirmed muscle-invasive bladder cancer (MIBC) who underwent radical cystectomy and had not received any prior treatment, whereas paired normal fibroblasts (NFs) were obtained from histologically normal bladder tissues at least 2 cm away from the tumor margin from the same patients. The Ethics Committee of the Affiliated Hospital of Qingdao University approved this study, and all participants provided informed consent. Fresh tissues were minced into ~ 1 mm³ fragments under sterile conditions and digested in collagenase type I (1 mg/mL, Solarbio) at 37 °C for 40 min. After digestion, the tissue suspension was gently triturated to obtain a single-cell suspension and filtered through a 70-µm cell strainer. Cells were collected by centrifugation and resuspended in DMEM (HyClone, #SH30249.02) supplemented with 10% fetal bovine serum (FBS; HyClone, #SH30406.05), 1% penicillin–streptomycin (HyClone, #SV30010), and a mycoplasma removal reagent (Yeasen, #40607ES). Cells were plated into culture dishes and maintained at 37 °C with 5% CO₂, with medium changes every 24–48 h. When cell confluence reached 80–90%, fibroblasts were passaged. Cells at passage 2 were cryopreserved, and CAFs at passages 2–6 were used for all experiments to ensure consistency [27]. Fibroblast purity was confirmed by immunofluorescence, showing expression of α-SMA and FAP and absence of epithelial (Pan-cytokeratin) and endothelial (CD31) markers.

### BLCA cell lines

The human BLCA cell lines T24 and 5637 were obtained from the Cell Bank of the Chinese Academy of Sciences (Shanghai, China). Cells were maintained in DMEM supplemented with 10% FBS and 1% penicillin–streptomycin and cultured at 37 °C in a humidified incubator with 5% CO₂.

### Stable transduction of CAFs

Lentiviral vectors used for stable transduction of CAFs were obtained from Genechem (China). CAFs were seeded one day prior to infection to achieve 30–50% confluence at the time of transduction. All lentiviral constructs carried a puromycin resistance gene for subsequent selection. Cells were infected in serum-free DMEM at a multiplicity of infection (MOI) of 10, and the medium was replaced with complete DMEM after 16–20 h. At 48 h post-infection, puromycin (5 µg/mL; Beyotime Biotechnology, #ST551) was added for continuous selection over 5–7 days to establish stable cell lines. Information on all viral vectors, including sequence details, is provided in Supplementary Tables 1 and Supplementary Table 2.

### Reagents

TGF-β1 (#HY-P7118), the γ-secretase inhibitor DAPT (#HY-13027), the protein synthesis inhibitor cycloheximide (CHX, #HY-12320), the proteasome inhibitor MG132 (#HY-13259), and the lysosomal inhibitor chloroquine (CQ, #HY-17589 A) were purchased from MedChemExpress (USA). The TGF-β/Smad pathway inhibitors SB-431,542 (#SF7890) and SD-208 (#SF7908), as well as the antioxidant N-acetyl-cysteine (NAC, #ST1546), were obtained from Beyotime Biotechnology (China).

### Quantitative real-time PCR (qPCR)

Total RNA was extracted using the AG RNAex Pro Reagent (Accurate Biotechnology, #AG21101, China). cDNA synthesis was performed with the Evo M-MLV RT Mix Kit (Accurate Biotechnology, #AG11728) according to the manufacturer’s instructions. Quantitative real-time PCR was carried out on a LightCycler 480 II system (Roche, Switzerland), with GAPDH used as the internal normalization control. Relative gene expression levels were calculated using the 2^^−ΔΔCt^ method. Primer sequences for all genes are listed in Supplementary Table 3.

### Immunoprecipitation and western blotting

Immunoprecipitation (IP) was performed using a commercial IP kit (Beyotime, #P2179) following the manufacturer’s protocol. Briefly, cells were lysed in IP lysis buffer supplemented with 1% protease inhibitor cocktail. Equal amounts of protein lysates were incubated with the indicated primary antibodies together with Protein A/G magnetic beads at 4 °C overnight with gentle rotation. Beads were washed at least three times with lysis buffer and eluted with 1× SDS loading buffer to obtain IP samples.

For Western blotting, whole-cell lysates were prepared in RIPA buffer (Beyotime, #P0013D) and quantified using the BCA Protein Quantification Kit (Thermo Fisher Scientific, #71285-M). Equal amounts of protein lysates or IP eluates were resolved by SDS–PAGE and transferred onto PVDF membranes (Millipore, USA). Membranes were blocked in 5% non-fat milk in TBST for 1 h at room temperature, followed by incubation with primary antibodies at 4 °C overnight. After washing, membranes were incubated with HRP-conjugated secondary antibodies for 1 h at room temperature. Signals were developed using enhanced chemiluminescence reagents (Thermo Fisher Scientific). Primary antibodies and working dilutions were as follows: NOTCH3 (1:1000, Proteintech, #55114-1-AP), α-SMA (1:20000, Proteintech, #67735-1-Ig), COL1A1 (1:1000, Cell Signaling Technology, #72026), SQSTM1/P62 (1:5000, Proteintech, #18420-1-AP), p-SMAD2/3 (1:1000, Cell Signaling Technology, #8828), SMAD2/3 (1:1000, Cell Signaling Technology, #5678), NRF2 (1:2000, Proteintech, #16396-1-AP), NOX4 (1:1000, Proteintech, #14347-1-AP), HO-1 (1:5000, Proteintech, #10701-1-AP), Ubiquitin (1:1000, Cell Signaling Technology, #3936), K48-linked polyubiquitin (1:1000, Cell Signaling Technology, #4289), HSPA8 (1:2000, Proteintech, #10654-1-AP), and GAPDH (1:1000, Cell Signaling Technology, #2118).

### ChIP–qPCR

ChIP–qPCR was performed using the Pierce™ Magnetic ChIP Kit (Thermo Scientific, #26157, USA) following the manufacturer’s optimized procedure with minor adaptations. Briefly, CAFs were cross-linked in 1% formaldehyde for 10 min, quenched with 125 mM glycine for 5 min, and washed twice with ice-cold PBS. Cell pellets were lysed sequentially to isolate nuclei, which were subjected to micrococcal nuclease digestion to obtain chromatin fragments of approximately 200–500 bp. Nuclei were then disrupted by brief sonication to release soluble chromatin. For immunoprecipitation, equal amounts of chromatin were incubated overnight at 4 °C with ChIP-grade antibody against SMAD2(Selleck, #E24M24) or normal Rabbit IgG (Cell Signaling Technology, #3900), followed by capture with Protein A/G magnetic beads. After sequential washes with low-salt and high-salt buffers, protein–DNA complexes were eluted and subjected to reverse crosslinking at 65 °C, followed by RNase A and Proteinase K digestion. DNA was purified using the supplied spin columns. Purified DNA was analyzed by qPCR using primers targeting the predicted SMAD2-binding region within the NOTCH3 promoter regions. ChIP enrichment was quantified using the %10 Input method and normalized to IgG controls. Primer sequences are listed in Supplementary Table 4.

### EdU incorporation assay

EdU incorporation assays were performed using the BeyoClick™ EdU-594 Cell Proliferation Kit (Beyotime, #C0078S) according to the manufacturer’s instructions. Cells were seeded into culture plates and allowed to adhere overnight before receiving the indicated treatments. Subsequently, cells were incubated with 10 µM EdU for 2 h at 37 °C in a 5% CO₂ incubator to allow incorporation into newly synthesized DNA. Following incubation, cells were fixed with 4% paraformaldehyde for 15 min, washed with PBS, and permeabilized using 0.3% Triton X-100 for 15 min at room temperature. The incorporated EdU was then detected using the kit-supplied Click reaction cocktail, applied for 30 min in the dark. Nuclei were counterstained with Hoechst 33,342 (1 µg/mL). Fluorescence images were acquired using an inverted fluorescence microscope. The percentage of EdU-positive cells (red fluorescence) relative to total nuclei (blue fluorescence) was quantified as a measure of cellular proliferative activity.

### Tumorsphere formation assay

T24 or 5637 BLCA cells were seeded into ultra-low attachment 6-well plates (Corning, #3471, USA) at a density of 1 × 10⁴ cells per well. Cells were cultured in serum-free DMEM/F12 medium supplemented with 20 ng/mL human recombinant EGF (MedChemExpress, #HY-P7109), 10 ng/mL human recombinant bFGF (MedChemExpress, #HY-P7004), 5 mg/mL insulin (Solarbio, #I8040), and 2% B-27 supplement (Gibco, #17504044). Culture medium was refreshed every 3 days. After 7 days, tumorspheres were imaged under an inverted microscope, and spheres with a diameter greater than 75 μm were quantified. The number and size of tumorspheres were used as indicators of cancer stem-like properties.

### Co-culture systems

#### Direct co-culture

Direct coculture of CAFs and BLCA cells was performed on chamber slides (24-well) precoated with Matrigel (BD Biosciences). Briefly, 50 µL of Matrigel diluted 1:10 in serum-free medium was added to each well and allowed to polymerize for 30 min at 37 °C. CAFs and tumor cells (T24 or 5637) were then mixed at a ratio of 5000:1000 and seeded onto the Matrigel-coated wells. Cells were maintained for 5 days under standard culture conditions before analysis. To distinguish the two cell populations, immunofluorescence staining was performed using antibodies against Pan-Keratin (tumor cells; 1:200, Cell Signaling Technology, #4545) and COL1A1 (fibroblasts; 1:200, Cell Signaling Technology, #72026). Images were acquired via fluorescence microscopy, and the expansion area of tumor cells was quantified using ImageJ.

#### Indirect coculture (conditioned medium)

For indirect coculture, CAFs were cultured in 10-cm dishes until they reached 80–90% confluence. Cells were then washed once with PBS and incubated in serum-free medium for 24 h to generate conditioned medium (CM). The collected CM was centrifuged to remove debris, supplemented with 10% fresh serum, and applied to cultured BLCA cells (T24 or 5637) for 5 days. Tumor cells treated with CM were subsequently subjected to downstream assays.

### Collagen gel contraction assay

Collagen gel contraction assays were performed using rat tail Collagen I (Corning, #354236). Briefly, 5 × 10⁵ CAFs were resuspended in 1 mL of collagen solution that had been mixed with complete DMEM at a 1:1 ratio. The collagen–cell mixture was evenly seeded into 12-well plates and incubated at 37 °C with 5% CO₂ for 1 h to allow gel polymerization. After polymerization, 1 mL of complete DMEM was gently added to each well. Using a 200-µL pipette tip, the gel was carefully detached from the well wall to enable free-floating contraction. Gels were then incubated for an additional 24 h, after which the extent of contraction was imaged and quantified. Gel area was measured using ImageJ and expressed as the percentage of area reduction relative to the initial gel size.

### CCK-8 assay

Cell proliferation was assessed using the CCK-8 assay (MedChemExpress, #HY-K0301). Cells were seeded into 96-well plates at a density of 1000 cells/well, with 5 technical replicates per condition. After cell attachment, the indicated treatments were applied. Cells were monitored daily for 5 consecutive days. At each time point, 10 µL of CCK-8 reagent was added to each well and incubated for 2 h at 37 °C with 5% CO₂. The absorbance at 450 nm (OD450) was measured using a microplate reader. Proliferation curves were generated based on the daily OD450 values to evaluate cell growth under different experimental conditions.

### Transwell migration and invasion assays

Cell migration and invasion were evaluated using Transwell chambers with 8.0-µm pore polycarbonate membranes (Corning, #3422). For migration assays, the upper chambers were pre-equilibrated with serum-free medium, and 5 × 10⁴ cells suspended in 200 µL serum-free medium were seeded into each upper chamber. The lower chambers were filled with 500 µL complete medium containing 10% FBS to serve as a chemoattractant. For invasion assays, the upper surface of the membrane was precoated with 100 µL of Matrigel (MedChemExpress, #HY-K6002) that had been diluted 1:8 in serum-free medium, and incubated at 37 °C for 60 min to allow polymerization. Cells were then seeded at the same density and cultured under the same conditions as in the migration assay. After 24 h of incubation at 37 °C with 5% CO₂, non-migrated or non-invaded cells remaining on the upper surface were gently removed with a cotton swab. Inserts were washed with PBS, fixed with 4% paraformaldehyde for 15–20 min, and stained with 0.1% crystal violet for 15–20 min. Excess dye was removed with PBS, and the membranes were air-dried. Cells that had migrated or invaded to the lower membrane surface were imaged under a light microscope in multiple randomly selected fields and quantified.

### Colony formation assay

Colony formation ability was assessed using a plate colony formation assay. Cells were seeded into 6-well plates at a density of 500 cells per well, gently distributed evenly, and cultured at 37 °C with 5% CO₂ for 10–14 days until visible colonies had formed. The medium was removed, and cells were washed once with PBS followed by fixation with 4% paraformaldehyde for 15 min. Fixed cells were stained with 0.1% crystal violet for 15–20 min, and excess dye was rinsed off with PBS before air-drying. Colonies measuring ≥ 1 mm in diameter were imaged using a microscope or scanner and quantified as an indicator of clonogenic capacity.

### ROS detection

Intracellular reactive oxygen species (ROS) levels were measured using the Reactive Oxygen Species Assay Kit (Beyotime, #S0036S). CM-DCFH-DA was diluted 1:1000 in probe dilution buffer to obtain a final working concentration of 5 µM. After cells reached the appropriate confluency, the culture medium was removed and replaced with sufficient CM-DCFH-DA working solution to fully cover the cells. Cells were incubated for 30 min at 37 °C in the dark under 5% CO₂. Following incubation, the dye was removed, and cells were gently washed with PBS to eliminate excess probe. Nuclei were counterstained with Hoechst 33,342, after which fluorescence images were captured immediately using an inverted fluorescence microscope. ROS levels were quantified based on CM-H2DCF-DA fluorescence intensity.

### JC-1 staining assay

Mitochondrial membrane potential was assessed using the JC-1 probe (MedChemExpress, #HY-15534) following the manufacturer’s instructions. Briefly, cells were seeded into 12-well plates and allowed to reach the appropriate confluency. After the indicated treatments, the culture medium was removed and replaced with freshly prepared JC-1 working solution diluted in serum-free medium to fully cover the cells. Cells were incubated for 30 min at 37 °C in the dark under 5% CO₂, followed by gentle washing with pre-warmed buffer to remove excess dye. Fluorescence images were acquired immediately using a fluorescence microscope. JC-1 aggregates (red fluorescence) indicate polarized mitochondria, whereas JC-1 monomers (green fluorescence) reflect mitochondrial depolarization. Mitochondrial membrane potential was quantified by calculating the red/green fluorescence intensity ratio using ImageJ.

### LC–MS/MS analysis

CAF cells were collected by gentle scraping and lysed using IP lysis buffer. Protein samples were separated by SDS–PAGE, stained with Coomassie Brilliant Blue, and the corresponding gel bands were excised for in-gel digestion with trypsin. The resulting peptides were analyzed by LC–MS/MS on a Thermo Scientific Q Exactive HF-X mass spectrometer equipped with a nano-ESI source. The spray voltage was set to 2.0 kV. MS1 spectra were acquired at a resolution of 60,000 over a mass range of 350–1500 m/z. The most intense precursor ions were subjected to higher-energy collisional dissociation (HCD), and MS2 spectra were acquired at a resolution of 15,000. Data were collected in a data-dependent acquisition (DDA) mode with an AGC target of 3 × 10^6^ for MS1, 1 × 10^5^ for MS2, and a dynamic exclusion window of 30 s. Raw MS/MS data were processed using Proteome Discoverer (v2.4) and searched against the UniProt human protein database. Trypsin/P was specified as the digestion enzyme, allowing up to two missed cleavages. The minimum peptide length was set to six amino acids. Carbamidomethylation of cysteine was included as a fixed modification, whereas methionine oxidation and N-terminal acetylation were set as variable modifications.

### Proximity ligation assay (PLA)

Protein–protein interactions in cells were examined using the NaveniFlex Cell Kit (Navinci Diagnostics AB, #60025) following the manufacturer’s instructions. Briefly, CAFs were seeded onto BeyoGold™ 35-mm glass-bottom confocal dishes (Beyotime, #FCFC016), fixed, and permeabilized. Cells were then blocked with Naveni Block at 37 °C for 1 h. Primary antibodies diluted according to the manufacturer’s recommendations were applied and incubated at 37 °C for 1 h or overnight at 4 °C, followed by thorough washing with TBS-T. Subsequently, Navenibody probes diluted 1:40 were added and incubated at 37 °C for 1 h, followed by washing. The Naveni Enzyme 1 reaction solution was then applied and incubated at 37 °C for 60 min, washed, and followed by incubation with the Naveni Enzyme 2 reaction solution for 90 min at 37 °C. Nuclei were counterstained with DAPI, and cells were rehydrated in PBS before imaging under a fluorescence microscope. Positive PLA signals were interpreted as indicative of close proximity and interaction between the target proteins.

### Mouse BLCA models

#### Subcutaneous xenograft model

All animal experiments were conducted in accordance with the ethical obligations and animal care guidelines approved by Qingdao University. Female BALB/c nude mice (4–6 weeks old; Beijing Vital River Laboratory Animal Technology) were anesthetized with inhaled isoflurane. A freshly prepared suspension of T24 or 5637 BLCA cells mixed with CAFs at a 1:1 ratio (final concentration, 1 × 10^8^ cells/mL) was gently injected subcutaneously into the left inguinal region (100 µL per mouse). Tumor growth and overall health status were assessed weekly, and tumor volume was calculated using the formula V = 0.5 × length × width^2^. At the study endpoint, mice were euthanized, and tumors were carefully excised, weighed, and fixed in 4% paraformaldehyde before paraffin embedding and histopathological evaluation.

#### Orthotopic BLCA model

For orthotopic implantation, female BALB/c nude mice (4–6 weeks old) were anesthetized with isoflurane and positioned supine. After standard abdominal sterilization, a small lower midline incision (~ 1 cm) was made to expose the bladder. A mixed suspension of T24 or 5637 cells and CAFs (1:1 ratio, 1 × 10^8^ cells/mL) was prepared immediately before implantation, and 10 µL was slowly delivered into the bladder wall using a microsyringe, taking care to avoid perforation or leakage. The muscle and skin layers were then sutured, and mice were allowed to recover before being housed under SPF conditions. Body weight and general condition were monitored throughout the experiment. Tumor development was followed longitudinally using an IVIS imaging system. At the endpoint, mice were euthanized, and the entire bladder was harvested, weighed, fixed, and processed for histological and immunohistochemical analyses.

### Sirius red, H&E and IHC staining

Paraffin-embedded tissue sections were deparaffinized in xylene for 5–10 min, repeated once, followed by rehydration through a graded ethanol series (100%, 90%, 80%, and 70%) and rinsing in distilled water. Sirius Red staining was performed using a commercial staining kit (Beyotime, #C0190). Sections were first stained with hematoxylin for approximately 10 min, differentiated in acid alcohol for 30 s, and blued under running tap water for 10 min. After rinsing in distilled water, 50 µL of Sirius Red solution was applied to each section for 10–15 min. Excess dye was removed by rapid washing in distilled water. Sections were then dehydrated through graded ethanol (70%, 80%, 90%, and 100%), cleared in xylene, and mounted with neutral resin. Under bright-field microscopy, collagen fibers appear red, nuclei dark purple, and muscle fibers yellow.

Hematoxylin and eosin (H&E) staining was performed using a standard kit (Beyotime, #C0105). Sections were stained in hematoxylin for 5–10 min, rinsed and blued for 10 min, briefly differentiated, and counterstained with eosin for 30 s–2 min. After dehydration through graded ethanol and clearing in xylene, sections were mounted with neutral resin. Nuclei appear blue, while cytoplasm and extracellular matrix display pink to red staining.

For immunohistochemistry, paraffin sections were deparaffinized, rehydrated, and subjected to antigen retrieval. Endogenous peroxidase activity was quenched using 3% H₂O₂. Sections were blocked with 5% BSA for 30 min and incubated with primary antibodies overnight at 4 °C. After PBS washing, HRP-conjugated secondary antibodies were applied, followed by DAB chromogenic development and hematoxylin counterstaining. Slides were dehydrated, cleared, mounted, and examined under a light microscope.

### Immunofluorescence staining (IF)

Immunofluorescence staining was performed using the BeyoTSA™ fluorescence staining kit following the manufacturer’s instructions. Cells or tissue sections were fixed with 4% paraformaldehyde for 15 min, washed with PBS, and permeabilized with 0.1% Triton X-100 for 5 min. Samples were then incubated with blocking buffer at room temperature for 1 h. Primary antibodies diluted at the recommended concentrations were applied and incubated for 1–2 h at room temperature. After washing, HRP-conjugated secondary antibodies were added and incubated for 30–60 min, followed by incubation with the TSA working solution for signal amplification in the dark for 10 min. Sections were then washed thoroughly. For dual staining, enhanced antibody stripping buffer was applied for 10 min at room temperature after the first round of TSA development to remove non–covalently bound antibody complexes, and the staining procedure was repeated for the second target. Finally, nuclei were counterstained with DAPI, and slides were mounted with antifade medium. Images were acquired using a fluorescence microscope.

The primary antibody dilutions were as follows: NOTCH3 (1:100, Proteintech, #55114-1-AP), α-SMA (1:200, Proteintech, #67735-1-Ig), SQSTM1/P62 (1:400, Proteintech, #66184-1-Ig), COL1A1 (1:200, Cell Signaling Technology, #72026), CK-6 (1:50, Abcam, #EPR1602Y), and Pan-Keratin (1:200, Cell Signaling Technology, #4545).

### RNA sequencing and data processing

RNA sequencing, including library construction and high-throughput sequencing, was performed by Genefund Biotech (Shanghai, China). In detail, total RNA was extracted from CAF samples and subjected to quality assessment to ensure an A260/A280 ratio of 1.8–2.1 and sufficient RNA quantity. Polyadenylated mRNA was enriched using oligo-dT magnetic beads, followed by controlled fragmentation and first- and second-strand cDNA synthesis. Sequencing libraries were generated using standard Illumina workflows, including end repair, 3′-A tailing, adaptor ligation, PCR amplification, and size selection (300–400 bp). Qualified libraries were sequenced on the Illumina NovaSeq 6000 platform to produce paired-end reads. Raw reads were processed by removing adaptor sequences and low-quality bases using Cutadapt and Trimmomatic. Clean reads were evaluated with FastQC to assess sequence quality metrics such as Q20/Q30 and GC content. High-quality reads (MAPQ ≥ 30) were aligned to the human reference genome (hg38, Ensembl v101) using HISAT2. Transcript assembly and quantification were performed with StringTie to obtain gene- and transcript-level expression profiles. Gene-level read counts were extracted using the prepDE.py script provided by StringTie. Differential expression analysis was conducted using limma, with paired-sample modeling applied where appropriate. Genes were considered expressed when ≥ 50% of samples in either group exhibited FPKM > 0.1. Functional enrichment for differentially expressed genes was performed using clusterProfiler for KEGG pathway analysis.

### Transcriptomic analysis

Publicly available CAF-related transcriptomic datasets were retrieved from the Gene Expression Omnibus (GEO), including GSE229047 (5 NFs and 8 CAFs from germ-cell tumors), GSE296349 (12 paired NF/CAF samples from breast cancer), and GSE46824 (9 NFs and 11 metastatic CAFs from colorectal cancer). Differential expression of NOTCH3 between CAFs and NFs was evaluated using paired or unpaired two-tailed Student’s t-tests, selected according to the original study design (paired tests for matched NF/CAF samples; unpaired tests for independent sample sets). Fibroblast and CAF abundance across TCGA-BLCA bulk transcriptomes (408 BLCA cases with matched clinical outcome data) was estimated using four established deconvolution frameworks: MCP-counter, xCell, EPIC, and TIDE. MCP-counter [28] and xCell [29] were implemented with their default stromal gene signatures to compute fibroblast enrichment scores. EPIC (https://epic.gfellerlab.org) and TIDE (http://tide.dfci.harvard.edu) were used to derive CAF-associated stromal signatures according to its predefined modules. All analyses were performed on log_2_(TPM + 1)–normalized expression matrices unless otherwise indicated. In addition, the TCGA-BLCA cohort and GSE13507 cohort (165 BLCA cases with matched clinical outcome data) were used to assess the prognostic relevance of the NOTCH3-related transcriptional signature.

### Single-cell RNA sequencing (scRNA-seq)

Single-cell RNA-seq datasets used in this study were obtained from the SRA database under BioProject PRJNA662018 (8 BLCA tissues and 3 paired adjacent normal tissues) [9] and from the China National GeneBank (CNGB) database (CNP0000460, comprising 8 BLCA and 8 adjacent normal samples) [12]. Raw FASTQ files were processed using Cell Ranger v6.1 with default parameters to generate gene–barcode count matrices. Downstream analyses were performed in R (v4.1.3) using the Seurat (v5) package. Quality control filters were applied to exclude cells expressing < 1000 or > 6000 genes, cells with > 10% mitochondrial transcripts, and those with > 5% hemoglobin-related genes, to remove low-quality cells and dying/erythrocyte-contaminated populations. After quality control, unique molecular identifier (UMI) count matrices were log-normalized, and batch effects across samples were corrected using the Harmony algorithm. Dimensionality reduction was performed using principal component analysis (PCA), and the top 30 principal components (PCs), selected based on the Seurat ElbowPlot, were used for graph-based clustering with FindClusters at a resolution of 0.8. Clusters were visualized using Uniform Manifold Approximation and Projection (UMAP). Differentially expressed genes for each cluster were identified using the FindAllMarkers function.

Major cell populations were annotated based on canonical lineage markers reported in previous studies, including mast cells (TPSAB1, KIT), B cells (MS4A1, CD79A), plasma cells (IGLC2, IGLC3, IGHG1), myeloid cells (CD68, LYZ), fibroblasts (COL1A1, COL3A1, DCN), pericytes/smooth muscle cells (ACTA2, RGS5), endothelial cells (VWF, PLVAP), T cells (CD3D, CD3E), and epithelial cells (KRT19, KRT18, EPCAM). CAF subtypes were further defined according to established marker signatures, including myCAF (ACTA2, COL1A1), iCAF (IL11, LIF) and apCAF (CD74, HLA-DRA).

### Spatial transcriptomics

Spatial transcriptomic data from bladder cancer patients were obtained from the GEO database (GSE285715) and processed using Seurat (v5). Quality control was performed at the spot level based on sequencing depth and RNA complexity, excluding spots with fewer than 500 UMIs, fewer than 200 detected genes, or a mitochondrial transcript proportion exceeding 20%. The Spatial assay was normalized and variance-stabilized using SCTransform. To infer cell-type signals across spatial locations, a well-annotated single-cell RNA sequencing reference dataset from matched tissue and species was used. Both the reference scRNA-seq data and the spatial transcriptomic data were normalized using an identical SCTransform workflow. Cell-type labels were subsequently transferred to spatial spots using Seurat’s anchor-based integration framework, yielding probabilistic cell-type prediction scores for each spot. Prediction scores for individual cell types were extracted and used as proxy measures of relative cell-type abundance in downstream analyses. For gene–cell-type spatial colocalization analysis, expression levels of target genes were extracted from the SCTransform-normalized spatial data, ensuring compatibility with the layer-based data structure. Spearman rank correlation was used to assess spatial associations between gene expression and cell-type prediction scores. To account for technical confounding, multivariable linear regression models were further fitted, with gene expression as the dependent variable, cell-type prediction score as the primary predictor, and sequencing depth and mitochondrial transcript proportion included as covariates. All P values were adjusted for multiple testing using the Benjamini–Hochberg false discovery rate (FDR) correction. Given the inherent spatial autocorrelation of spatial transcriptomic data, permutation testing was performed to evaluate the robustness of observed associations. Specifically, cell-type prediction scores were randomly permuted across spatial spots while gene expression values were held constant (1,000 iterations), and empirical P values were calculated from the resulting null distributions of correlation coefficients, followed by FDR correction. Spatial expression patterns of genes and cell-type prediction scores were visualized using spatial feature maps, with scatter plots and correlation heatmaps used to summarize gene–cell-type associations. Gene–cell-type relationships that remained significant after both permutation-based FDR correction and multivariable regression adjustment were considered statistically robust.

### Statistical analysis

All statistical analyses were performed using R (v4.1.3) and GraphPad Prism (v10.2.0). As indicated in the figure legends, data are presented as mean ± SEM or mean ± SD, as appropriate. The statistical tests applied to each experiment are specified in the corresponding figure legends. Unless otherwise stated, comparisons between two groups were assessed using two-tailed Student’s t-tests. For analyses involving more than two groups, one-way ANOVA followed by Bonferroni’s post-hoc correction was used. A P value < 0.05 was considered statistically significant.

## Results

### CAF signature is enriched in BLCA and associated with poor clinical outcome

To systematically assess the biological features and clinical relevance of CAFs in bladder cancer, we first performed comparative transcriptomic profiling of patient-derived CAFs (*n* = 6) and matched NFs (*n* = 6). Differential expression analysis identified a large set of genes significantly upregulated in CAFs, including established markers of fibroblast activation and extracellular matrix remodeling, such as ACTA2, COL4A1, INHBA, and ANKRD1 (Figure S1A). Based on these upregulated genes (fold change > 2, *p* < 0.05), we generated a CAF signature for subsequent integrative analyses.

Analysis of single-cell RNA-sequencing data showed that this CAF signature was highly enriched in the fibroblast compartment, with minimal expression in epithelial, immune, and endothelial cells, supporting its cell-type specificity (Figure S1B–D). CAF signature scores were also significantly elevated in tumor tissues relative to normal tissues, indicating prominent CAF activation in the BLCA microenvironment (Figure S1C, D).

Clinically, higher CAF signature expression was associated with more advanced disease, including higher T stage, lymph node metastasis, advanced clinical stage, and high tumor grade, although no significant association was observed with distant metastasis (Figure S1E–J). Kaplan–Meier analyses further showed that high CAF signature expression was significantly associated with worse overall survival in the TCGA-BLCA cohort, and this finding was validated in the GSE13507 and GSE48075 cohorts (Figure S1K–M).

Collectively, these data indicate that BLCA CAFs display a distinct activated transcriptional program, and that the CAF signature is strongly associated with disease progression and poor clinical outcome.

### NOTCH3 is enriched in CAF within the BLCA microenvironment

**Fig. 1:**
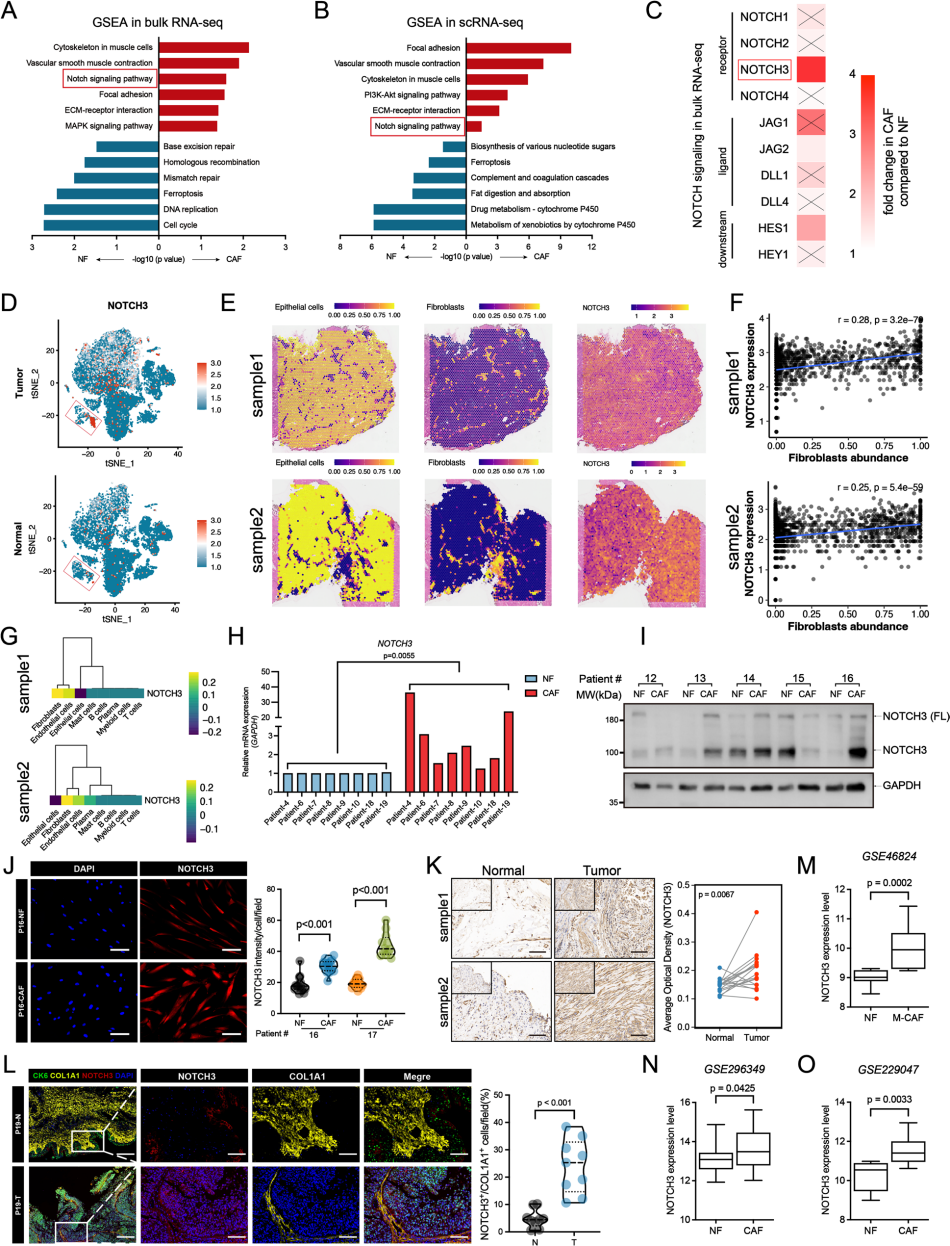
NOTCH3 is enriched in CAF within the bladder cancer microenvironment. **A**, **B**. Gene set enrichment analysis (GSEA) of bulk RNA-seq (**a**) and single-cell RNA-seq (**b**) datasets comparing CAFs and NFs, showing enrichment of stromal remodeling- and contractility-related pathways, including NOTCH signaling, in CAFs. **C**. Heatmap showing fold changes of NOTCH pathway components in CAFs relative to NFs based on bulk RNA-seq. **D**. t-SNE visualization of NOTCH3 expression in fibroblasts from tumor and adjacent normal tissues based on single-cell RNA-seq data. **E**. Spatial transcriptomic maps from two representative BLCA samples showing the spatial distribution of epithelial cells, fibroblasts, and NOTCH3 expression. **F**. Correlation analysis between fibroblast abundance and NOTCH3 expression across spatial transcriptomic spots. **G**. Hierarchical clustering showing preferential association of NOTCH3 expression with fibroblast populations across two spatial transcriptomic samples. **H**. RT-qPCR analysis of NOTCH3 mRNA expression in primary CAFs and matched NFs derived from BLCA patients. **I**. Immunoblot analysis of NOTCH3 protein expression in paired NF and CAF samples from five BLCA patients. **J**. Representative immunofluorescence images of NOTCH3 (red) and DAPI (blue) in primary NFs and CAFs from patients #16 and #17, with quantification of NOTCH3 fluorescence intensity per cell. Scale bar, 300 μm. **K**. Representative immunohistochemical images of NOTCH3 staining in stromal regions of paired normal and tumor BLCA tissues, with quantification of stromal NOTCH3 staining intensity. **L**. Multiplex immunofluorescence staining of normal and tumor tissues showing CK6 (epithelial marker, green), COL1A1 (fibroblast marker, yellow), NOTCH3 (red), and DAPI (blue), with quantification of the proportion of NOTCH3⁺COL1A1⁺ cells. Scale bars = 300 μm (left) and 75 μm (right). **M**–**O**. NOTCH3 expression in CAFs and NFs across independent transcriptomic datasets from colorectal cancer (GSE46824) (**m**), breast cancer (GSE296349) (**n**), and germ cell tumors (GSE229047) (**o**). Data are presented as mean ± SD. Statistical significance was assessed using two-tailed unpaired t-test (panels **L**, **M**, **N**, **O**), two-tailed paired t-test (panels **H**, **I**) or two-way ANOVA with Šidák’s post hoc multiple-comparison test (panel **K**)

To identify signaling pathways associated with CAFs in BLCA, we analyzed transcriptomic profiles of primary CAFs and matched NFs. Both bulk and single-cell RNA-sequencing analyses consistently highlighted activation of stromal remodeling and contractility-related programs in CAFs, including focal adhesion, vascular smooth muscle contraction, extracellular matrix–receptor interaction, and notably, the NOTCH signaling pathway (Fig. 1A, B). Among individual NOTCH pathway components, NOTCH3 was the only receptor significantly upregulated in CAFs, whereas other receptors, canonical ligands, and downstream effectors showed minimal or nonsignificant changes (Fig. 1C).

Further analyses supported a CAF enrichment pattern of NOTCH3. Single-cell visualization showed preferential NOTCH3 expression in CAFs from tumor tissues, while fibroblasts from adjacent normal tissues exhibited minimal expression (Fig. 1D). Spatial transcriptomic analysis likewise demonstrated that fibroblast-rich regions exhibited higher NOTCH3 expression, with significant positive correlations between fibroblast abundance and NOTCH3 levels across independent BLCA samples (Fig. 1E–G).

This pattern was validated in patient-derived fibroblasts and clinical specimens. Compared with matched NFs, CAFs showed significantly higher NOTCH3 expression at both the mRNA and protein levels, as confirmed by qPCR, immunoblotting, and immunofluorescence staining (Fig. 1H–J). In bladder cancer tissues, immunohistochemistry and multiplex immunofluorescence revealed elevated stromal NOTCH3 expression, predominantly in COL1A1⁺ fibroblasts within tumor regions, whereas COL1A1⁺ fibroblasts in adjacent normal tissues exhibited minimal expression (Fig. 1K–L), indicating a tumor-induced fibroblast activation state.

Finally, analysis of independent transcriptomic datasets from colorectal cancer, breast cancer, and germ cell tumors revealed a similar pattern of NOTCH3 upregulation in CAFs relative to NFs, suggesting that fibroblast-associated NOTCH3 enrichment is not limited to BLCA (Fig. 1M–O).

Collectively, these findings identify NOTCH3 as a CAF-enriched NOTCH receptor in BLCA, rather than a general fibroblast marker.

### CAF-intrinsic NOTCH3 promotes and amplifies tumor-supportive functions

**Fig. 2:**
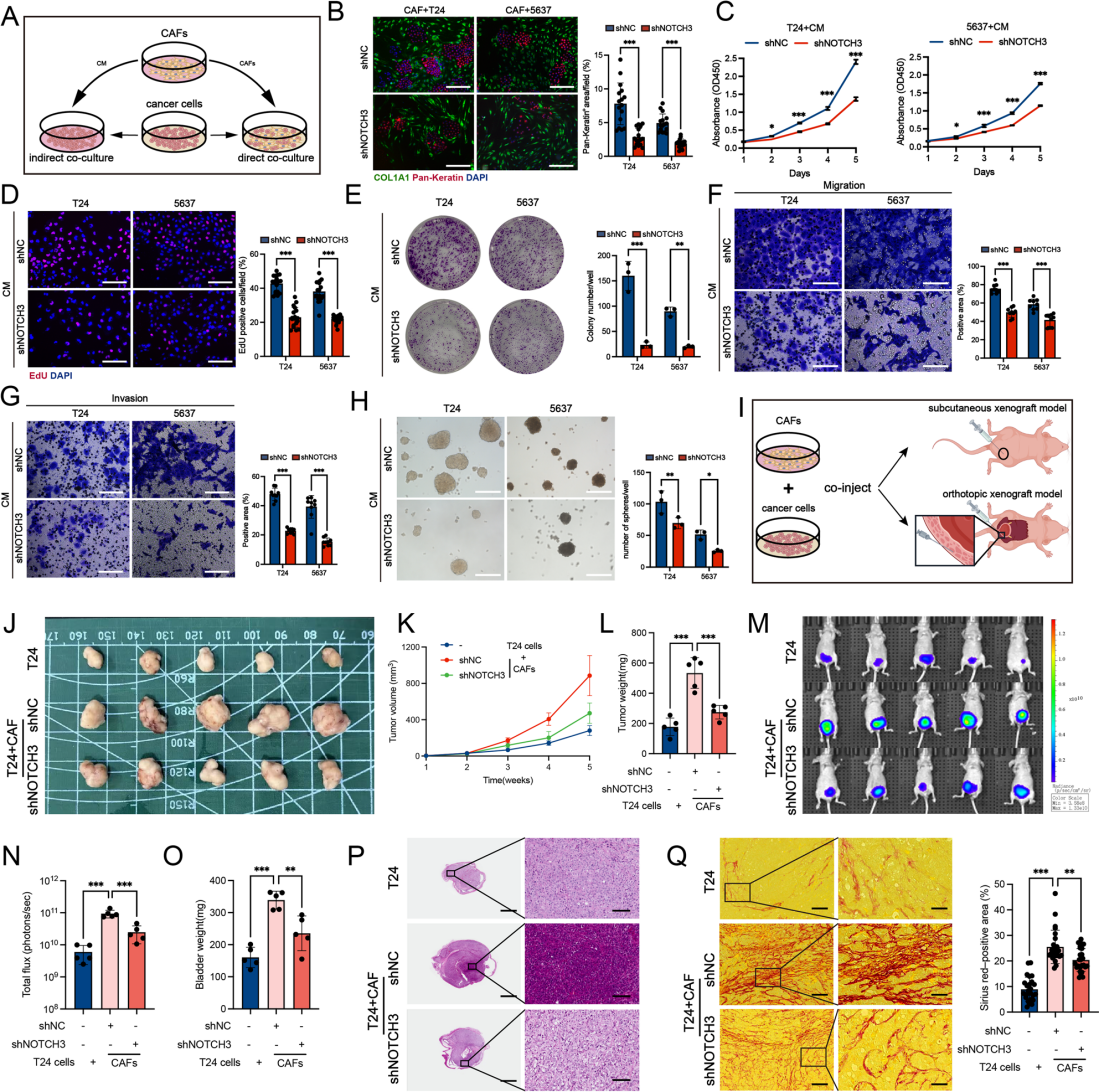
CAF-intrinsic NOTCH3 promotes and amplifies tumor-supportive functions. **A**. Schematic illustration of indirect co-culture using CAF-conditioned medium (CM) and direct co-culture systems used to evaluate the tumor-supportive effects of CAFs. **B**. Representative immunofluorescence images of direct co-culture assays showing Pan-Keratin-positive bladder cancer cell clusters (red), COL1A1-positive CAFs (green), and DAPI (blue), together with quantification of Pan-Keratin–positive area in T24 and 5637 cells co-cultured with shNC or shNOTCH3 CAFs. Scale bar = 300 μm. **C**. Growth curves of T24 and 5637 cells cultured with conditioned medium derived from shNC or shNOTCH3 CAFs. **D**. Representative EdU staining images and quantification of EdU-positive T24 and 5637 cells cultured with conditioned medium from shNC or shNOTCH3 CAFs. Scale bar = 300 μm. **E**. Representative colony formation assays and quantification of colony numbers in T24 and 5637 cells cultured with conditioned medium from shNC or shNOTCH3 CAFs. Scale bar = 300 μm. **F**. Representative Transwell migration assays and quantification of migrated T24 and 5637 cells cultured with conditioned medium from shNC or shNOTCH3 CAFs. Scale bar = 300 μm. **G**. Representative Transwell invasion assays and quantification of invaded T24 and 5637 cells cultured with conditioned medium from shNC or shNOTCH3 CAFs. Scale bar = 300 μm. **H**. Representative tumorsphere formation assays and quantification of sphere numbers in T24 and 5637 cells cultured with conditioned medium from shNC or shNOTCH3 CAFs. Scale bar = 75 μm. **I**. Schematic of subcutaneous and orthotopic co-injection xenograft models established using bladder cancer cells and CAFs. **J**. Representative images of subcutaneous tumors formed by T24 cells alone or co-injected with shNC or shNOTCH3 CAFs. **K**. Tumor growth curves of subcutaneous xenografts. **L**. Endpoint tumor weights of subcutaneous xenografts. **M**. Representative bioluminescence images of orthotopic xenograft models. **N**. Quantification of total photon flux in orthotopic xenografts. **O**. Endpoint bladder weights in orthotopic xenografts. **P**. Representative hematoxylin–eosin (H&E) staining of orthotopic tumors. Scale bar = 3 mm (left) and 80 μm (right). **Q**. Representative Sirius Red staining and quantification of Sirius Red–positive area in orthotopic tumors. Scale bar = 150 μm (left) and 37.5 μm (right). Data are presented as mean ± SD. Statistical significance was assessed using two-way ANOVA with Šidák’s multiple-comparison test (panels **B**, **D**–**H**), two-tailed unpaired Student’s t test (panel **C**) and one-way ANOVA with Dunnett’s post hoc multiple-comparison test (panels **L**, **N**, **O**, **Q**). *P < 0.05, **P < 0.01, ***P < 0.001

Given the established tumor-promoting role of CAFs in BLCA, which can drive tumor progression via both direct cell–cell interactions and paracrine signaling, we examined whether elevated NOTCH3 expression in CAFs is functionally required for these effects using direct co-culture and conditioned medium (CM) based systems (Fig. 2A).

In direct co-culture assays, immunofluorescence staining of the epithelial marker Pan-Keratin revealed a marked reduction in cancer cell cluster expansion when co-cultured with shNOTCH3 CAFs compared with control CAFs, indicating impaired contact-dependent tumor–stroma interactions (Fig. 2B). Similarly, conditioned medium derived from shNOTCH3 CAFs significantly suppressed proliferation of T24 and 5637 cells, as reflected by reduced growth kinetics and diminished EdU incorporation (Fig. 2C, D). In addition, long-term clonogenic capacity was markedly impaired, as demonstrated by decreased colony formation efficiency (Fig. 2E). In addition, conditioned medium from NOTCH3-silenced CAFs significantly attenuated the migratory and invasive capacities of T24/5637 cells (Fig. 2F, G). Furthermore, tumorsphere formation assays revealed a marked reduction in sphere-forming efficiency following exposure to NOTCH3-knockdown CAF-conditioned medium, indicating impaired tumor cell self-renewal capacity (Fig. 2H).

To determine whether these effects extend to in vivo tumor progression, we established both subcutaneous and orthotopic xenograft models by co-injecting BLCA cells with control or NOTCH3-silenced CAFs (Fig. 2I).

Tumors formed in the presence of shNOTCH3 CAFs exhibited markedly reduced growth kinetics, tumor volume, and tumor weight compared with controls (Fig. 2J–L). Bioluminescence imaging in the orthotopic model further confirmed significantly diminished tumor burden upon co-injection with NOTCH3-knockdown CAFs (Fig. 2M–O). While CAF co-injection markedly enhances tumor growth compared with tumor cells alone, NOTCH3 knockdown consistently attenuates this effect, indicating that NOTCH3 functions to potentiate CAF-mediated tumor-promoting activity. Histopathological analyses revealed decreased stromal expansion and attenuated collagen deposition in tumors generated with shNOTCH3 CAFs, as evidenced by hematoxylin–eosin and Sirius Red staining (Fig. 2P–Q).

Collectively, these data demonstrate that CAF-intrinsic NOTCH3 is required to maintain tumor-supportive functions in both in vitro and in vivo contexts in BLCA.

### NOTCH3 maintains the myofibroblastic phenotype of CAFs

**Fig. 3:**
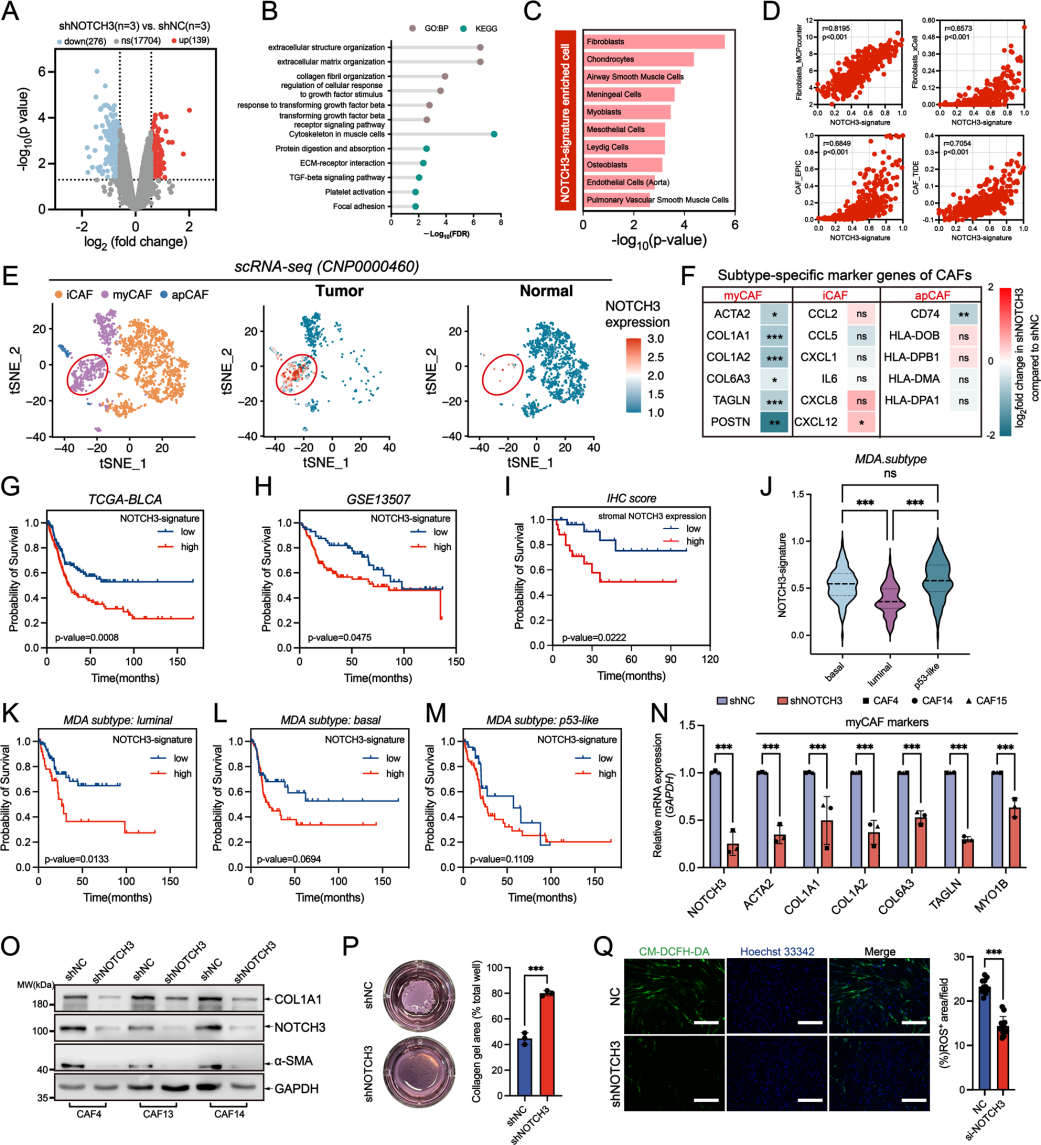
NOTCH3 maintains the myofibroblastic phenotype of CAFs. **A**. Volcano plot showing differentially expressed genes in primary CAFs following NOTCH3 knockdown (shNOTCH3 vs. shNC; |fold change| > 1.5, P < 0.05). **B**. GO and KEGG pathway enrichment analyses of differentially expressed genes after NOTCH3 silencing. **C**. Cell-type enrichment analysis of genes significantly downregulated upon NOTCH3 knockdown (|fold change| > 2, P < 0.05). **D**. Correlation between the NOTCH3-associated transcriptional signature and fibroblast/CAF abundance scores in the TCGA-BLCA cohort estimated by MCP-counter, xCell, EPIC, and TIDE. **E**. t-SNE visualization of CAF subpopulations (myCAF, iCAF, and apCAF) from the public single-cell RNA-seq dataset CNP0000460. **F**. Heatmap showing the effects of NOTCH3 knockdown on subtype-specific marker genes of myCAF, iCAF, and apCAF populations. **G**, **H**. Kaplan–Meier analyses of overall survival stratified by NOTCH3-signature expression in the TCGA-BLCA (**g**) and GSE13507 (**h**) cohorts. **I**. Kaplan–Meier analysis of overall survival stratified by stromal NOTCH3 immunohistochemical staining scores in clinical BLCA specimens. **J**. Distribution of NOTCH3-signature scores across molecular subtypes defined by the MD Anderson classification. **K**–**M**. Kaplan–Meier analyses of overall survival stratified by NOTCH3-signature expression within luminal (**k**), basal (**l**), and p53-like (**m**) subtypes. **N**. RT–qPCR analysis of NOTCH3 and myCAF marker genes in shNC and shNOTCH3 CAFs. **O**. Immunoblot analysis of NOTCH3 and myCAF markers (COL1A1 and α-SMA) in shNC and shNOTCH3 CAFs. **P**. Collagen gel contraction assay and quantification of gel contraction area following NOTCH3 knockdown in CAFs. **Q**. Representative CM-DCFH-DA staining and quantification of ROS-positive area in CAFs after NOTCH3 silencing. Scale bar = 300 μm. Data are presented as mean ± SD. Statistical significance was assessed using Pearson correlation analysis (panel **D**), the log-rank test (panels **G**–**I**, **K**-**M**), ordinary one-way ANOVA with Tukey’s post hoc multiple-comparisons test (panel J) and two-tailed unpaired Student’s t test (panels **N**, **P**, **Q**). *P < 0.05, **P < 0.01, ***P < 0.001

Having established that CAF-intrinsic NOTCH3 is required for tumor-supportive functions, we next sought to delineate the underlying molecular programs through which NOTCH3 regulates CAF identity. To this end, we performed bulk RNA sequencing following NOTCH3 knockdown in primary CAFs (Fig. 3A).

Differential expression analysis identified widespread transcriptional reprogramming upon NOTCH3 knockdown. GO and KEGG pathway enrichment analyses revealed significant suppression of extracellular matrix organization, collagen fibril formation, focal adhesion, cytoskeletal remodeling, and TGF-β signaling pathways (Fig. 3B), indicating disruption of core contractile and matrix-associated programs characteristic of activated CAFs. To further characterize NOTCH3-dependent transcriptional regulation, genes significantly altered in shNOTCH3 CAFs (|FC| > 2, *p* < 0.05) were defined as a NOTCH3-signature. Cell-type enrichment analysis demonstrated that these regulated genes were predominantly associated with fibroblast and mesenchymal lineages (Fig. 3C). Consistently, multiple independent deconvolution algorithms—including MCP-counter, xCell, EPIC, and TIDE—revealed strong positive correlations between the NOTCH3-associated signature and fibroblast/CAF abundance across BLCA transcriptomes (Fig. 3D), supporting fibroblast-specific enrichment in bulk tumor datasets.

Single-cell RNA-seq analyses further demonstrated preferential enrichment of NOTCH3 expression within the myCAF subpopulation compared with inflammatory (iCAF) or antigen-presenting (apCAF) subsets (Fig. 3E, Figure S2A), indicating subtype specificity. At the gene level, silencing NOTCH3 resulted in pronounced downregulation of canonical myCAF markers, including ACTA2, COL1A1, COL1A2, COL6A3, TAGLN, and POSTN, whereas genes associated with iCAF or apCAF states exhibited minimal or nonsignificant changes (Fig. 3F). Clinically, high expression of the NOTCH3-associated signature was significantly associated with poorer overall survival in both the TCGA-BLCA and GSE13507 cohorts (Fig. 3G–H), and elevated stromal NOTCH3 expression similarly correlated with adverse patient outcomes (Fig. 3I). The MD Anderson Cancer Center molecular classification stratifies BLCA into basal, luminal, and p53-like subtypes with distinct clinical implications [30, 31]. We applied this classification to evaluate the clinical relevance of NOTCH3 activation. The NOTCH3-signature was enriched in basal and p53-like subtypes compared with luminal tumors (Fig. 3J). Moreover, subtype-stratified survival analysis revealed that the NOTCH3 signature effectively stratified prognosis in luminal patients and showed a similar trend in basal tumors, whereas no significant prognostic impact was observed in the p53-like subtype (Fig. 3K–M), suggesting that the clinical impact of NOTCH3 varies across molecular subtypes.

At the molecular level, qPCR, immunoblotting, and immunofluorescence analyses confirmed that NOTCH3 regulates the expression of myCAF markers (Fig. 3N–O, Figure S2B). Functionally, NOTCH3 knockdown significantly impaired collagen gel contraction (Fig. 3P), confirming loss of contractile myofibroblastic activity. Given the established contribution of reactive oxygen species (ROS) to stabilization of myCAF phenotypes, we next assessed intracellular redox status. NOTCH3 knockdown significantly reduced ROS accumulation in CAFs and partially restored mitochondrial membrane potential, as assessed by JC-1 staining (Fig. 3Q, Figure S2C).

Collectively, these findings demonstrate that NOTCH3 sustains transcriptional, structural, and redox programs that define the myCAF phenotype.

### NOTCH3 is directly transcriptionally regulated by TGF-β/SMAD2 signaling

**Fig. 4:**
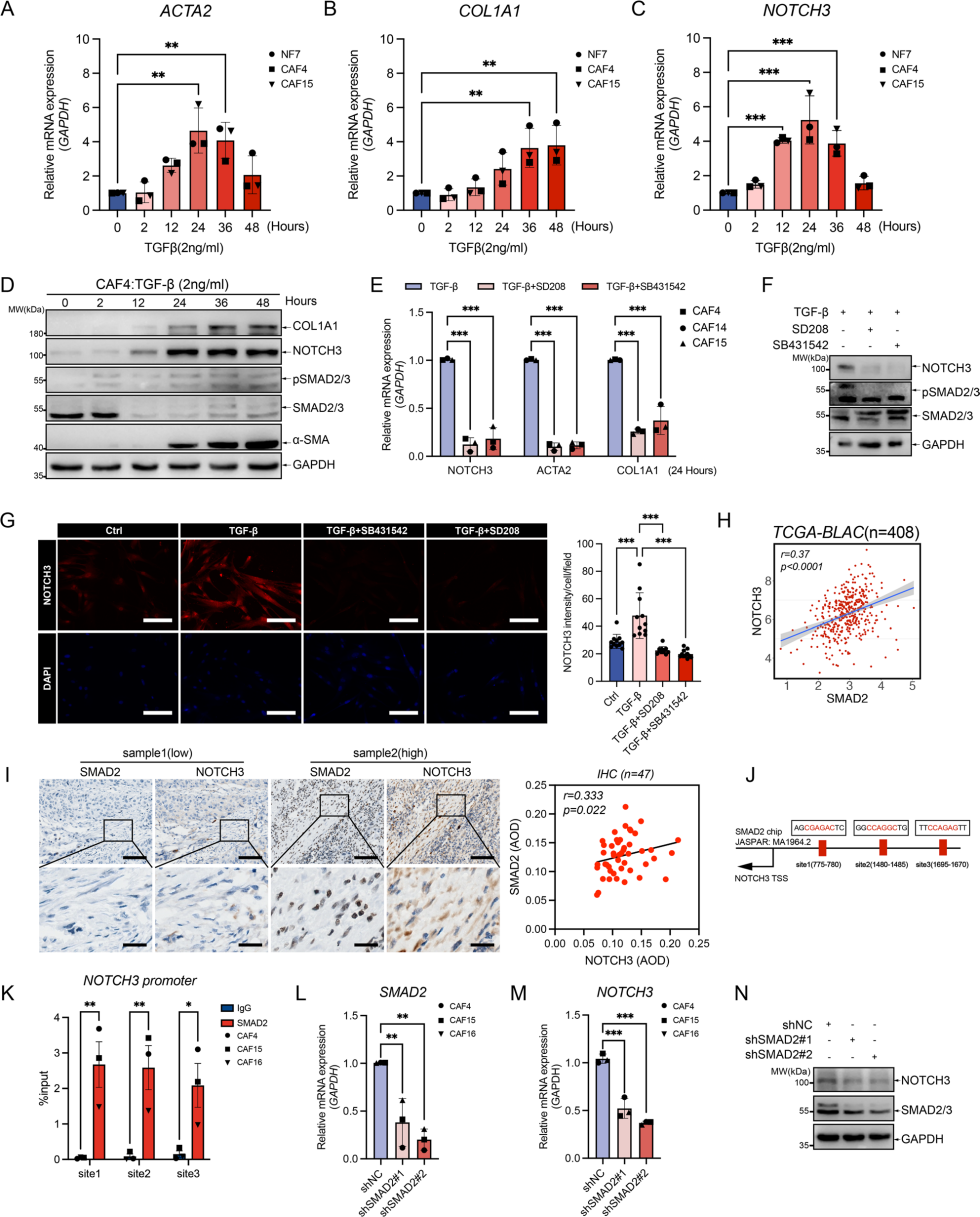
NOTCH3 is transcriptionally regulated by TGF-β/SMAD2 signaling. **A**–**C**. Time-course RT–qPCR analysis of ACTA2 (**A**), COL1A1 (**B**), and NOTCH3 (**C**) mRNA expression in primary CAFs/NFs following stimulation with recombinant TGF-β1 (2 ng/mL) for the indicated time points. **D**. Immunoblot analysis of CAF4 treated with TGF-β1 (2 ng/mL) for the indicated time points. **E**. RT–qPCR analysis of NOTCH3, ACTA2, and COL1A1 expression in CAFs treated with TGF-β1 alone or in combination with the TGF-β receptor inhibitors SD208 or SB431542 for 24 h. **F**. Immunoblot analysis showing the effects of SD208 and SB431542 on TGF-β1–induced NOTCH3 expression in CAF4. **G**. Representative immunofluorescence images and quantification of NOTCH3 staining intensity in CAFs treated with TGF-β1 in the presence or absence of SD208 or SB431542. Scale bar = 300 μm. **H**. Correlation analysis between SMAD2 and NOTCH3 mRNA expression in the TCGA-BLCA cohort (n = 408). **I**. Representative immunohistochemical images of stromal SMAD2 and NOTCH3 staining in bladder cancer tissues with low or high expression levels, together with correlation analysis between stromal SMAD2 and NOTCH3 staining intensities (IHC cohort, n = 47). Scale bar = 100 μm (top) and 33 μm (bottom). **J**. Schematic of predicted SMAD2 binding sites within the NOTCH3 promoter identified using the JASPAR database (https://jaspar.elixir.no). **K**. ChIP–qPCR analysis showing SMAD2 enrichment at the indicated sites within the NOTCH3 promoter in CAFs. IgG served as a negative control. **L**, **M**. RT–qPCR analysis of SMAD2 (**L**) and NOTCH3 (**M**) mRNA expression in CAFs transduced with two independent SMAD2-targeting shRNAs. **N**. Immunoblot analysis confirming reduced NOTCH3 protein expression following SMAD2 knockdown. Data are presented as mean ± SD. Statistical significance was assessed using one-way ANOVA with Dunnett’s post hoc multiple-comparison test (panels **A**–**C**, **G**, **L**, **M**), two-way ANOVA with Šidák’s multiple-comparison test (panels **E**, **K**) and Spearman correlation analysis (panels H, I). *P < 0.05, **P < 0.01, ***P < 0.001

Transcriptomic analyses showed that NOTCH3 knockdown suppressed cytoskeletal, focal adhesion, extracellular matrix, and TGF-β–related programs in CAFs (Fig. 3B), prompting us to examine whether TGF-β signaling regulates NOTCH3 expression. Stimulation of CAFs with recombinant TGF-β1 (2 ng/mL) induced a time-dependent increase in NOTCH3 mRNA and protein levels, accompanied by upregulation of the myCAF markers ACTA2 and COL1A1 (Fig. 4A–D, Figure S3A). Consistent with this observation, conditioned medium derived from T24 bladder cancer cells (T24-CM) similarly induced NOTCH3 expression in CAFs (Figure S3B–D), indicating that tumor-derived signals can activate this pathway.

Inhibition of TGF-β receptors with SB431542 or SD208 markedly suppressed TGF-β–induced expression of NOTCH3 and myCAF markers at both mRNA and protein levels (Fig. 4E–G). Similar inhibitory effects were observed when CAFs were stimulated with T24-CM in the presence of these inhibitors (Figure S3E), supporting receptor-dependent regulation.

Because SMAD2 functions as a key transcriptional mediator downstream of canonical TGF-β signaling, we next examined its relationship with NOTCH3. NOTCH3 expression positively correlated with SMAD2 mRNA levels in the TCGA-BLCA cohort (Fig. 4H). A similar positive association was observed at the protein level in BLCA stromal regions based on immunohistochemical staining (Fig. 4I).

Motif analysis identified three putative SMAD2 binding sites within the NOTCH3 promoter (Fig. 4J), and ChIP-qPCR confirmed significant SMAD2 enrichment at these sites (Fig. 4K). Furthermore, silencing SMAD2 significantly reduced NOTCH3 mRNA and protein expression in CAFs (Fig. 4L–N, Figure S3F–G).

Together, these findings indicate that canonical TGF-β signaling promotes NOTCH3 expression through SMAD2-dependent transcriptional regulation.

### NOTCH3 promotes ROS accumulation and reinforces the myCAF phenotype by suppressing P62

**Fig. 5:**
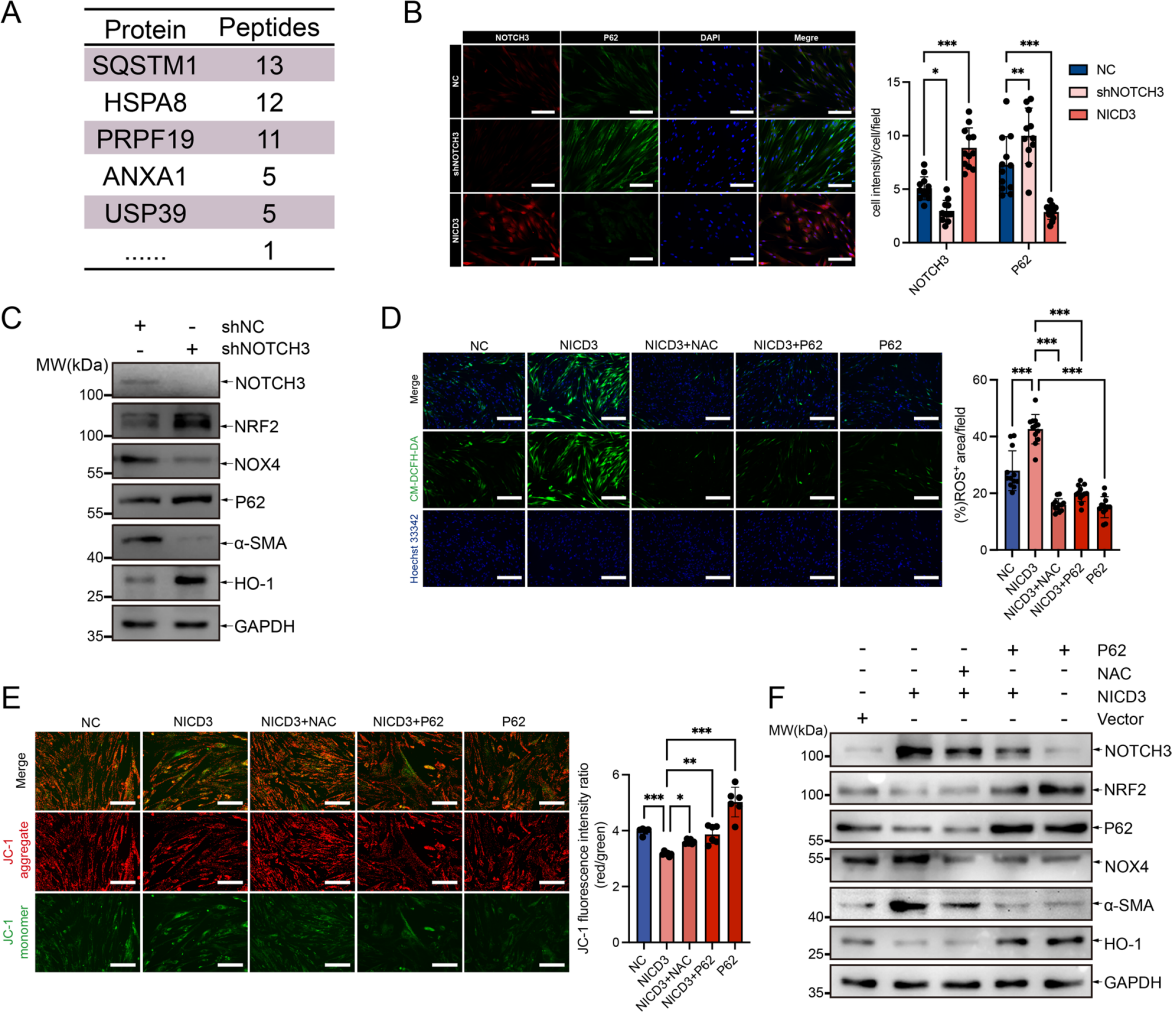
NOTCH3 promotes ROS accumulation and reinforces the myCAF phenotype by suppressing P62. **A**. Identification of NOTCH3-interacting proteins by immunoprecipitation followed by mass spectrometry (IP–MS). **B**. Representative immunofluorescence images showing NOTCH3 (red), P62 (green) and DAPI (blue) in CAFs transfected with negative control (NC), shNOTCH3 or NICD3. Quantification of NOTCH3 and P62 fluorescence intensity per cell is shown on the right. Scale bars, 300 μm. **C**. Immunoblot analysis of CAFs transiently transfected with shNOTCH3 or shNC. GAPDH served as a loading control. **D**. Representative CM-DCFH-DA fluorescence images of CAFs expressing NICD3, with or without NAC treatment or P62 rescue. Quantification (right) shows the ROS-positive area per field. Scale bars, 300 μm. **E**. JC-1 staining of mitochondrial membrane potential in CAFs expressing NICD3, with or without NAC treatment or P62 rescue. Representative images (left) and quantification of JC-1 aggregate/monomer ratios (right). Scale bars, 150 μm. **F**. Immunoblot validation of NICD3 overexpression and rescue experiments. GAPDH served as a loading control. Data are presented as mean ± SD. Statistical significance was determined by two-way ANOVA with Šidák’s post hoc multiple-comparison test (panel **B**) and one-way ANOVA with post hoc multiple-comparison testing (panels D, E), *P < 0.05, **P < 0.01, ***P < 0.001

Having established that NOTCH3 sustains redox features of the myCAF phenotype, we next investigated the molecular basis underlying this effect. IP–MS analysis identified SQSTM1/P62 as one of the top NOTCH3-associated candidate proteins (Fig. 5A). Given that P62 is a key regulator of oxidative stress homeostasis and is closely linked to NRF2-dependent antioxidant signaling [32–36], we asked whether NOTCH3 promotes ROS accumulation through modulation of the P62-associated redox pathway.

NOTCH3 knockdown increased P62, NRF2, and HO-1, while reducing NOX4 and α-SMA (Fig. 5B, C), supporting activation of an antioxidant program and attenuation of the myCAF state.

We then asked whether NOTCH3 activation exerts the opposite effect. NICD3 overexpression markedly increased intracellular ROS and reduced mitochondrial membrane potential, as shown by CM-DCFH-DA and JC-1 staining (Fig. 5D–E). Importantly, either P62 re-expression or ROS scavenging with NAC largely reversed these changes. At the molecular level, both interventions restored P62, NRF2, and HO-1, while suppressing NOX4 and α-SMA in NICD3-expressing CAFs (Fig. 5B, F). Immunofluorescence analysis showed that NOTCH3 and P62 were co-localized in CAFs (Figure S4).

Together, these results indicate that NOTCH3 promotes ROS accumulation and reinforces the myCAF phenotype, at least in part, by suppressing the P62-associated antioxidant program.

### NOTCH3 promotes proteasomal degradation of P62 through HSPA8-mediated K48 ubiquitination

**Fig. 6:**
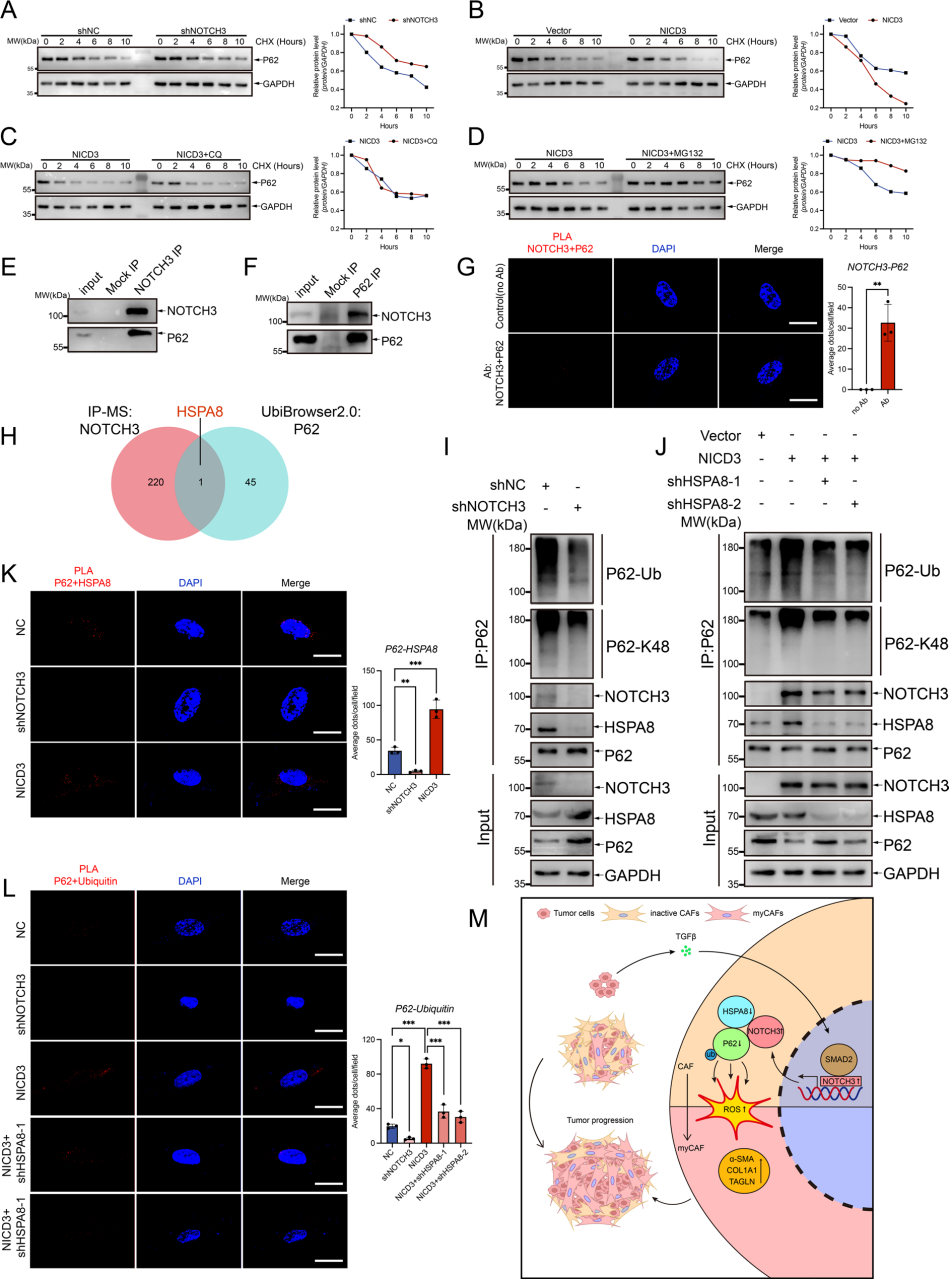
NOTCH3 promotes proteasomal degradation of P62 through HSPA8-mediated K48 ubiquitination. **A**, **B**. Cycloheximide (CHX) chase assays assessing P62 protein stability in CAFs with NOTCH3 knockdown (**A**) or NICD3 overexpression (**B**). **C**, **D**. Pharmacological inhibition of distinct degradative pathways in NICD3-overexpressing CAFs. Lysosomal blockade with chloroquine (CQ) failed to prevent NICD3-driven P62 loss (**C**), whereas the proteasome inhibitor MG132 effectively restored P62 abundance (**D**), indicating that NOTCH3-induced P62 degradation is primarily proteasome-dependent. **E**, **F**. Co-immunoprecipitation analyses demonstrating the physical interaction between NOTCH3 and P62 in CAFs. **G**. Representative proximity ligation assay (PLA) images showing the spatial interaction between endogenous NOTCH3 and P62 in CAFs. Quantification (right) indicates the average number of NOTCH3–P62 PLA puncta per cell. Scale bars: 20 μm. **H**. Integration of NOTCH3 IP–MS data with ubiquitin-network predictions (UbiBrowser 2.0, http://ubibrowser.bio-it.cn) identifies HSPA8 as both a NOTCH3-interacting protein and a putative ubiquitination-associated regulator of P62. **I**, **J**. P62 immunoprecipitation followed by immunoblotting to assess total and K48-linked ubiquitination under NOTCH3 knockdown (**I**) or NICD3 overexpression with/without HSPA8 silencing (**J**). **K**. Representative PLA images showing the interaction between HSPA8 and P62 in CAFs under NC, si-NOTCH3 or NICD3 overexpression conditions. Quantification (right) shows the average number of HSPA8–P62 PLA puncta per cell. Scale bars, 20 μm. **L**. Representative PLA images showing P62–ubiquitin interactions in CAFs under the indicated conditions. Quantification (right) indicates the average number of P62–ubiquitin PLA puncta per cell. Scale bars, 20 μm. **M**. Schematic illustration summarizing the proposed NOTCH3–HSPA8–P62 regulatory axis. Data are presented as mean ± SD. Statistical significance was assessed using two-tailed unpaired t-test (panel **G**) or one-way ANOVA with Dunnett’s post hoc multiple-comparison test (panels **K**, **L**). *P < 0.05, **P < 0.01, ***P < 0.001

To further define how NOTCH3 regulates P62 stability, we first examined the degradation kinetics of P62. CHX chase assays showed that NOTCH3 knockdown slowed P62 turnover, whereas NICD3 overexpression accelerated its degradation (Fig. 6A–B). To determine the responsible degradative route, we blocked lysosomal or proteasomal pathways pharmacologically. CQ failed to rescue the reduction of P62 induced by NICD3 (Fig. 6C), whereas MG132 markedly restored P62 abundance (Fig. 6D), indicating that NOTCH3 promotes proteasome-dependent degradation of P62.

We next examined whether NOTCH3 physically associates with P62. Co-immunoprecipitation assays demonstrated reciprocal association between NOTCH3 and P62 (Fig. 6E–F), and PLA further confirmed their close spatial proximity in CAFs (Fig. 6G).

To identify potential cofactors involved in this process, we integrated NOTCH3 IP–MS data with ubiquitination predictions from UbiBrowser2.0. HSPA8 emerged as the only shared candidate, appearing both in the NOTCH3 interactome and among predicted regulators of P62 ubiquitination (Fig. 6H). Consistent with this prediction, PLA analysis showed that the proximity between P62 and HSPA8 was reduced by NOTCH3 silencing and enhanced by NICD3 overexpression (Fig. 6K), suggesting that NOTCH3 facilitates recruitment of HSPA8 to P62.

We then examined the ubiquitination status of P62. NOTCH3 knockdown reduced both total and K48-linked ubiquitination of P62 (Fig. 6I), whereas NICD3 overexpression increased P62 ubiquitination and K48 linkage (Fig. 6J). Importantly, silencing HSPA8 abolished the increase in P62 ubiquitination induced by NICD3 (Fig. 6J). Consistently, PLA analysis confirmed that P62–ubiquitin proximity was diminished by NOTCH3 depletion, enhanced by NICD3, and reversed by HSPA8 knockdown (Fig. 6L).

Together, these results indicate that NOTCH3 recruits HSPA8 to promote K48-linked ubiquitination of P62, thereby accelerating its proteasomal degradation. This mechanism provides the molecular basis by which NOTCH3 lowers P62 abundance and sustains ROS-associated myCAF phenotypes (Fig. 6M).

### In vivo validation of the NOTCH3–HSPA8–P62 axis in bladder cancer

**Fig. 7:**
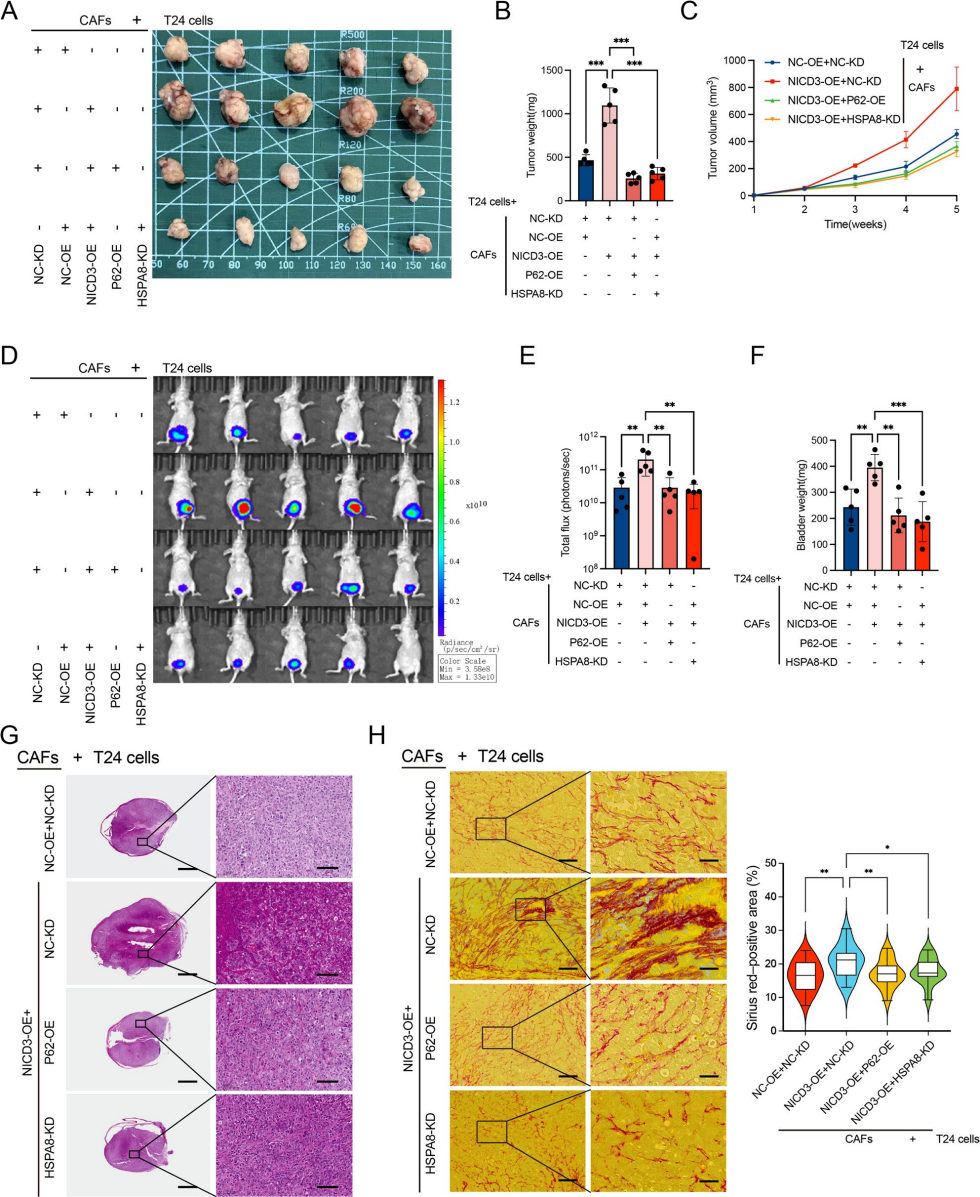
In vivo validation of the NOTCH3–HSPA8–P62 axis in bladder cancer. **A**. Representative images of tumors excised from subcutaneous xenograft models generated by co-injecting T24 BLCA cells with CAFs subjected to the indicated genetic manipulations (NC-OE + NC-KD, NICD3-OE + NC-KD, NICD3-OE+P62-OE, and NICD3-OE+HSPA8-KD). **B**. Quantification of tumor weights from the subcutaneous xenograft model. **C**. Tumor growth curves showing tumor volume progression in the indicated groups over time. **D**. Representative bioluminescence imaging of orthotopic bladder tumor models established by co-implanting T24-luc cells with genetically modified CAFs. **E**. Quantification of total bioluminescent flux from orthotopic tumors. **F**. Final bladder weights from orthotopic tumor-bearing mice in the indicated groups. **G**. Representative hematoxylin and eosin (H&E) staining of tumor sections derived from the orthotopic model, showing overall tumor architecture and cellular morphology. **H**. Sirius Red staining of tumor tissues to evaluate collagen deposition and stromal remodeling. Quantification of Sirius Red–positive areas is shown on the right. Data are presented as mean ± SD. Statistical significance was assessed using one-way ANOVA with Dunnett’s post hoc multiple-comparison test (panels **B**, **E**, **F**, **H**). *P < 0.05, **P < 0.01, ***P < 0.001

To determine whether the tumor-promoting effects of CAF-intrinsic NOTCH3 depend on the HSPA8/P62 axis in vivo, we established subcutaneous and orthotopic co-implantation models using T24 cells together with CAFs carrying different genetic perturbations, including control CAFs, NICD3-OE CAFs, NICD3-OE CAFs with P62-OE, and NICD3-OE CAFs with HSPA8-KD (Fig. 7A).

In the subcutaneous model, co-injection of T24 cells with NICD3-OE CAFs markedly increased tumor growth, as reflected by enlarged tumor size, increased tumor weight, and accelerated tumor volume expansion compared with the control group (Fig. 7A–C). Importantly, re-expression of P62 or knock down of HSPA8 substantially attenuated the tumor-promoting effect of NICD3-OE CAFs, indicating that both P62 restoration and HSPA8 suppression are sufficient to blunt NOTCH3-driven tumor enhancement in vivo.

Consistent results were obtained in the orthotopic model. Bioluminescence imaging showed that tumors formed in the presence of NICD3-OE CAFs displayed markedly increased signal intensity, whereas P62-OE or HSPA8-KD significantly reduced tumor burden (Fig. 7D–E). Bladder weights at endpoint showed the same pattern, further supporting a requirement for the HSPA8/P62 axis in mediating the protumorigenic activity of NOTCH3 in CAFs (Fig. 7F).

Histological analyses provided additional support for this conclusion. Hematoxylin–eosin staining showed that NICD3-OE CAFs promoted more extensive tumor growth, whereas this effect was attenuated by P62-OE or HSPA8-KD (Fig. 7G). Sirius Red staining further demonstrated that NICD3 activation in CAFs enhanced collagen deposition and stromal fibrosis, while both P62-OE and HSPA8-KD significantly reduced matrix accumulation in vivo (Fig. 7H).

Together, these findings demonstrate that the tumor-promoting and pro-fibrotic effects of CAF-intrinsic NOTCH3 in vivo are largely dependent on the HSPA8/P62 axis.

## Discussion

CAFs exert essential regulatory functions within the BLCA microenvironment; however, the molecular mechanisms driving their heterogeneity and functional diversification remain incompletely defined [8, 37–39]. The myCAF subset—characterized by strong tumor-promoting and fibrogenic properties—has emerged as a central yet unresolved component of tumor stroma [15, 40, 41]. Here, through integrated multi-omics analysis, functional assays, and protein interaction studies, we delineate a regulatory cascade involving the TGF-β/SMAD2–NOTCH3–HSPA8–P62–ROS axis. This framework connects extracellular signaling, transcriptional regulation, protein homeostasis, and redox balance into a unified mechanism governing CAF phenotypic specification, providing a coherent model for the formation and maintenance of myCAF.

We demonstrate that NOTCH3 is highly enriched in CAFs in BLCA and shows similar stromal upregulation across multiple tumor types, suggesting a conserved microenvironmental role. NOTCH3-associated transcriptional signatures correlate with poor prognosis in BLCA cohorts. While previous studies have focused on tumor cell–intrinsic NOTCH signaling, its CAF-specific functions have been underexplored. Through loss- and gain-of-function experiments, we identify NOTCH3 as an important regulator of myCAF differentiation, consistent with its role in fibrosis, tissue remodeling, and therapeutic resistance. This stromal-centered perspective expands current understanding of tumor microenvironment plasticity.

We further identify the upstream origin of NOTCH3 activation. TGF-β is a well-established driver of CAF activation, but how it selectively promotes myCAF differentiation has remained unclear [37]. Using SMAD2 ChIP assays, gene knockdown, and pharmacologic inhibition, we show that SMAD2 directly binds to the NOTCH3 promoter and activates its transcription. Consistently, immunohistochemical analysis of clinical specimens reveals a positive correlation between SMAD2 and NOTCH3 expression levels in stromal compartments, supporting the clinical relevance of this regulatory relationship. Together, these findings establish NOTCH3 as a direct effector of TGF-β/SMAD2 signaling and provide a mechanistic explanation for sustained myCAF activation. However, additional regulatory factors may also contribute to NOTCH3 expression, warranting further investigation.

At the downstream level, we identify ROS regulation as a central mechanism by which NOTCH3 controls CAF function. Previous studies have shown that NOTCH3 enhances ROS production in vascular smooth muscle cells [42]. Consistently, NOTCH3 knockdown reduces ROS levels and suppresses the myCAF phenotype, whereas NICD3 overexpression induces ROS accumulation. Mechanistically, NOTCH3 promotes ROS by destabilizing the antioxidant protein P62, thereby weakening redox buffering capacity and enhancing myCAF marker expression. These findings highlight oxidative stress as a key determinant of CAF fate, although additional ROS-independent pathways may also exist.

A major mechanistic advance is the identification of HSPA8 as a mediator of NOTCH3-induced P62 degradation. HSPA8 is known for its roles in protein folding and autophagy, but it can also function in ubiquitination processes [43, 44]. Previous studies show that HSPA8 cooperates with E3 ligases such as CHIP and SKP2 to promote K48-linked ubiquitination and proteasomal degradation [45, 46]. Our data demonstrate that NOTCH3 recruits HSPA8 to facilitate K48-linked ubiquitination of P62, leading to its proteasomal degradation. This finding reveals that CAF plasticity is regulated not only by transcriptional programs but also by dynamic protein turnover.

In vivo, suppression of NOTCH3 in CAFs significantly inhibits tumor growth and stromal remodeling. Compared with direct ROS targeting, NOTCH3 inhibition may represent a more effective strategy, as ROS is transient and buffered by antioxidant systems, limiting the efficacy of ROS-targeted therapies [44–46]. As an upstream regulator, NOTCH3 continuously drives P62 degradation and ROS accumulation, acting as a stable molecular hub that sustains the myCAF phenotype and its tumor-promoting functions. Thus, targeting NOTCH3 may simultaneously disrupt redox balance, ECM remodeling, and stromal support, offering greater translational potential. However, its safety and generalizability require further evaluation, particularly given the potential risks associated with CAF targeting [47].

In vivo, suppression of NOTCH3 in CAFs attenuates tumor growth and stromal remodeling. Notably, these effects are partial rather than complete, which is consistent with the functional heterogeneity of CAFs. CAFs comprise multiple subpopulations with distinct tumor-promoting mechanisms, and NOTCH3 appears to preferentially regulate the myCAF subtype. As an upstream regulator, NOTCH3 promotes P62 degradation and ROS accumulation, thereby sustaining the myCAF phenotype and its associated pro-tumorigenic functions.

The persistence of tumor growth and collagen deposition following NOTCH3 inhibition suggests that other CAF subsets, such as iCAFs or additional stromal populations, may contribute to tumor progression through NOTCH3-independent pathways. Therefore, the effects of NOTCH3 targeting are selective rather than universal, reflecting its role in subtype-specific regulation of CAF function.

Compared with direct ROS targeting, NOTCH3 inhibition may represent a more stable upstream intervention, as ROS is transient and buffered by antioxidant systems, limiting the efficacy of ROS-targeted therapies [46–48]. Thus, targeting NOTCH3 may preferentially disrupt myCAF-associated redox balance, ECM remodeling, and stromal support, offering a mechanistically defined and potentially more precise therapeutic strategy. However, its safety and generalizability require further evaluation, particularly given the complexity of CAF biology and the potential risks associated with CAF targeting [49].

Despite defining this regulatory axis, several limitations remain. First, our in vivo models rely on immunodeficient mice, limiting evaluation of immune-related effects. Second, although CAFs enhance tumor growth compared with normal fibroblasts, the tumor cell–intrinsic molecular changes induced by CAFs, particularly via NOTCH3 signaling, have not been systematically characterized. Third, although we identify NOTCH3 as a key regulator of myCAF phenotypes, its upstream regulatory cues and potential interactions with other signaling pathways in the tumor microenvironment remain to be further defined. Future studies using single-cell approaches will help address these questions and validate our findings across diverse tumor contexts.

## Supplementary Information

Supplementary Material 1.

## Data Availability

The data that support the findings of this study are available from the corresponding author upon reasonable request.

## Abbreviations

apCAFs: Antigen-presenting cancer-associated fibroblasts
BLCA: Bladder cancer
CAFs: Cancer-associated fibroblasts
ChIP: Chromatin immunoprecipitation
CHX: Cycloheximide
CM: Conditioned medium
CQ: Chloroquine
FBS: Fetal bovine serum
FDR: False discovery rate
H&E: Hematoxylin and eosin
IHC: Immunohistochemistry
IF: Immunofluorescence
iCAFs: Inflammatory cancer-associated fibroblasts
IP: Immunoprecipitation
irCAFs: Interferon-regulated cancer-associated fibroblasts
MIBC: Muscle-invasive bladder cancer
MOI: Multiplicity of infection
myCAFs: Myofibroblastic cancer-associated fibroblasts
NAC: N-acetyl-cysteine
NFs: Normal fibroblasts
PCA: Principal component analysis
PCs: Principal components
PLA: Proximity ligation assay
qPCR: Quantitative real-time PCR
RNA-seq: RNA sequencing
ROS: Reactive oxygen species
scRNA-seq: Single-cell RNA sequencing
UMI: Unique molecular identifier
