# Supplementary material for: NOTCH3 regulates myofibroblastic CAF differentiation via the P62–ROS signaling axis to promote bladder cancer progression
Source: J Exp Clin Cancer Res. 2026 Jun 6;45:173. doi: 10.1186/s13046-026-03751-1 (PMC13459780; doi:10.1186/s13046-026-03751-1)
Supplement: Supplementary file 1 [file 13046_2026_3751_MOESM1_ESM.docx]

**Supplementary materials**

**NOTCH3 Regulates Myofibroblastic CAF Differentiation via the P62–ROS Signaling Axis to Promote Bladder Cancer Progression**

Ding Hu, Chengquan Shen, Changxue Liu, Fei Xie, Xinning Wang, Hexiang Wang, Yunqing Chen, Xinzhao Zhao, Ruize Qin, Cheng Li, Huaixi Ge, Jianhang Zhu, Zhengze Xi, Yonghua Wang^*^

The supplementary materials includes:

1) Figure S1 to S4

2) Supplementary table 1 to 4

**Figure S1. CAF is enriched in BLCA and associated with poor clinical outcome.**

**A**. Volcano plot showing differentially expressed genes between patient-derived CAFs (n = 6) and matched NFs (n = 6). Genes with |log₂FC| > 1 and *P* < 0.05 are highlighted. **B**. t-SNE visualization of major cell populations identified from BLCA scRNA-seq data (CNP0000460). **C**. t-SNE plots showing CAF signature scores in tumor and adjacent normal samples derived from scRNA-seq data. **D**. Violin plots showing CAF signature scores across annotated cell types in tumor and normal samples. **E–J**. Violin plots of CAF signature scores stratified by patient age (≤65 vs. >65) (E), tumor T stage (T1–T4) (F), lymph node status (N0 vs. N1–3) (G), distant metastasis status (M0 vs. M1) (H), clinical stage (Stage I–IV) (I) and tumor grade (low vs. high) (J). **K–M**. Kaplan–Meier analyses of overall survival stratified by CAF signature expression in the TCGA-BLCA (K), GSE13507 (L) and GSE48075 (M) cohorts. Data are presented as median with interquartile range. Statistical significance was assessed using two-tailed Wilcoxon rank-sum test (panels E–J) or log-rank test (panels K–M).

**Figure S2. NOTCH3 maintains the myofibroblastic phenotype of CAFs.**

**A**. Analysis of a published single-cell RNA-seq dataset (PRJNA662018) showing the distribution of CAF subtypes (myCAF, iCAF, and apCAF) in bladder cancer tissues. Feature plots illustrate NOTCH3 expression across CAF populations and between tumor and normal samples, with higher expression enriched in the myCAF cluster. **B**. Immunofluorescence staining of primary CAFs transduced with control shRNA (shNC) or shRNA targeting NOTCH3 (shNOTCH3). Cells were stained for NOTCH3 (red), α-SMA (green), and DAPI (blue). Representative images (left) and quantification (right) of fluorescence intensity per field are shown, demonstrating reduced α-SMA expression following NOTCH3 knockdown. Scale bar = 300 μm. **C**. JC-1 staining to evaluate mitochondrial membrane potential in CAFs with or without NOTCH3 knockdown. Representative images (left) of JC-1 monomers (green) and aggregates (red) and the quantified red/green fluorescence ratio (right) are shown. Scale bar = 150 μm. Data are presented as mean ± SD. Statistical significance was assessed using two-way ANOVA with Šidák’s multiple-comparison test (panel B). **P* < 0.05, ***P* < 0.01, ****P* < 0.001.

**Figure S3. NOTCH3 is transcriptionally regulated by TGF-β/SMAD2 signaling.**

**A**. Immunoblot analysis of CAF15 cells treated with recombinant TGF-β (2 ng/mL) for the indicated time points (0–48 h). GAPDH served as a loading control. **B**. RT–qPCR analysis of NOTCH3, ACTA2, and COL1A1 mRNA expression in normal fibroblasts (NF7) and CAFs (CAF4 and CAF15) treated with T24 cell–derived conditioned medium (T24-CM) for 24 h. Data are normalized to GAPDH and presented relative to the 0 h group. **C, D**. Immunoblot analysis of NOTCH3 expression in CAF4 (C) and CAF15 (D) cells following treatment with T24 cell–derived conditioned medium for the indicated time points. **E**. Immunoblot analysis of CAF4 cells treated with T24-CM in the presence or absence of the TGF-β receptor inhibitor SD208 or the SMAD3 inhibitor SB431542. **F, G**. Immunoblot analysis of CAF15 (F) and CAF16 (G) cells transduced with control shRNA (shNC) or shRNAs targeting SMAD2 (shSMAD2#1 and shSMAD2#2). GAPDH served as a loading control. Data are presented as mean ± SD. Statistical significance was assessed using two-way ANOVA with Šidák’s multiple-comparison test (panel B). **P* < 0.05, ***P* < 0.01, ****P* < 0.001.

**Figure S4. Co-localization of NOTCH3 and P62 in CAFs.**

Immunofluorescence staining of CAFs treated with the proteasome inhibitor MG132 shows the distribution of P62 (green), NOTCH3 (red), and nuclei (DAPI, blue). Merged images indicate substantial spatial overlap between NOTCH3 and P62 signals. Co-localization analysis was performed using Pearson’s correlation coefficient, yielding a high degree of correlation (R = 0.894), supporting the association of NOTCH3 with P62 in CAFs. Scale bars, 75 μm.

**Supplementary Table 1 | Target sequences of shRNAs used for gene silencing.**

| Symbol | Target sequence (5′–3′) |
| --- | --- |
| sh-NOTCH3 | CCACGAGGATGCTATCTGT |
| sh-SMAD2#1 | GGUGUUCGAUAGCAUAUUATT |
| sh-SMAD2#2 | CCCUGCAACAGUGUGUAAATT |
| sh-HSPA8#1 | CCACCAUUGAAGAGGUUGATT |
| sh-HSPA8#2 | CUUGAGUCCUAUGCCUUCATT |

**Supplementary Table 2 | Coding sequences of overexpression constructs.**

| Symbol | Coding sequence (5′–3′) |
| --- | --- |
| NICD3 | GTCCACTCCCAGGTCCAACTGCACCTCGGTTCTGCTAGCGTTTAAACGGGCCCTCTAGACGCCACCATGGCCCGGCGCAAGCGCGAGCACAGCACCCTCTGGTTCCCTGAGGGCTTCTCACTGCACAAGGACGTGGCCTCTGGTCACAAGGGCCGGCGGGAACCCGTGGGCCAGGACGCGCTGGGCATGAAGAACATGGCCAAGGGTGAGAGCCTGATGGGGGAGGTGGCCACAGACTGGATGGACACAGAGTGCCCAGAGGCCAAGCGGCTAAAGGTAGAGGAGCCAGGCATGGGGGCTGAGGAGGCTGTGGATTGCCGTCAGTGGACTCAACACCATCTGGTTGCTGCTGACATCCGCGTGGCACCAGCCATGGCACTGACACCACCACAGGGCGACGCAGATGCTGATGGCATGGATGTCAATGTGCGTGGCCCAGATGGCTTCACCCCGCTAATGCTGGCTTCCTTCTGTGGGGGGGCTCTGGAGCCAATGCCAACTGAAGAGGATGAGGCAGATGACACATCAGCTAGCATCATCTCCGACCTGATCTGCCAGGGGGCTCAGCTTGGGGCACGGACTGACCGTACTGGCGAGACTGCTTTGCACCTGGCTGCCCGTTATGCCCGTGCTGATGCAGCCAAGCGGCTGCTGGATGCTGGGGCAGACACCAATGCCCAGGACCACTCAGGCCGCACTCCCCTGCACACAGCTGTCACAGCCGATGCCCAGGGTGTCTTCCAGATTCTCATCCGAAACCGCTCTACAGACTTGGATGCCCGCATGGCAGATGGCTCAACGGCACTGATCCTGGCGGCCCGCCTGGCAGTAGAGGGCATGGTGGAAGAGCTCATCGCCAGCCATGCTGATGTCAATGCTGTGGATGAGCTTGGGAAATCAGCCTTACACTGGGCTGCGGCTGTGAACAACGTGGAAGCCACTTTGGCCCTGCTCAAAAATGGAGCCAATAAGGACATGCAGGATAGCAAGGAGGAGACCCCCCTATTCCTGGCCGCCCGCGAGGGCAGCTATGAGGCTGCCAAGCTGCTGTTGGACCACTTTGCCAACCGTGAGATCACCGACCACCTGGACAGGCTGCCGCGGGACGTAGCCCAGGAGAGACTGCACCAGGACATCGTGCGCTTGCTGGATCAACCCAGTGGGCCCCGCAGCCCCCCCGGTCCCCACGGCCTGGGGCCTCTGCTCTGTCCTCCAGGGGCCTTCCTCCCTGGCCTCAAAGCGGCACAGTCGGGGTCCAAGAAGAGCAGGAGGCCCCCCGGGAAGGCGGGGCTGGGGCCGCAGGGGCCCCGGGGGCGGGGCAAGAAGCTGACGCTGGCCTGCCCGGGCCCCCTGGCTGACAGCTCGGTCACGCTGTCGCCCGTGGACTCGCTGGACTCCCCGCGGCCTTTCGGTGGGCCCCCTGCTTCCCCTGGTGGCTTCCCCCTTGAGGGGCCCTATGCAGCTGCCACTGCCACTGCAGTGTCTCTGGCACAGCTTGGTGGCCCAGGCCGGGCGGGTCTAGGGCGCCAGCCCCCTGGAGGATGTGTACTCAGCCTGGGCCTGCTGAACCCTGTGGCTGTGCCCCTCGATTGGGCCCGGCTGCCCCCACCTGCCCCTCCAGGCCCCTCGTTCCTGCTGCCACTGGCGCCGGGACCCCAGCTGCTCAACCCAGGGACCCCCGTCTCCCCGCAGGAGCGGCCCCCGCCTTACCTGGCAGTCCCAGGACATGGCGAGGAGTACCCGGCGGCTGGGGCACACAGCAGCCCCCCAAAGGCCCGCTTCCTGCGGGTTCCCAGTGAGCACCCTTACCTGACCCCATCCCCCGAATCCCCTGAGCACTGGGCCAGCCCCTCACCTCCCTCCCTCTCAGACTGGTCCGAATCCACGCCTAGCCCAGCCACTGCCACTGGGGCCATGGCCACCACCACTGGGGCACTGCCTGCCCAGCCACTTCCCTTGTCTGTTCCCAGCTCCCTTGCTCAGGCCCAGACCCAGCTGGGGCCCCAGCCGGAAGTTACCCCCAAGAGGCAAGTGTTGGCCGGAGGTGGAGGATCAATGTCCCCTATACTAGGTTATTGGAAAATTAAGGGCCTTGTGCAACCCACTCGACTTCTTTTGGAATATCTTGAAGAAAAATATGAAGAGCATTTGTATGAGCGCGATGAAGGTGATAAATGGCGAAACAAAAAGTTTGAATTGGGTTTGGAGTTTCCCAATCTTCCTTATTATATTGATGGTGATGTTAAATTAACACAGTCTATGGCCATCATACGTTATATAGCTGACAAGCACAACATGTTGGGTGGTTGTCCAAAAGAGCGTGCAGAGATTTCAATGCTTGAAGGAGCGGTTTTGGATATTAGATACGGTGTTTCGAGAATTGCATATAGTAAAGACTTTGAAACTCTCAAAGTTGATTTTCTTAGCAAGCTACCTGAAATGCTGAAAATGTTCGAAGATCGTTTATGTCATAAAACATATTTAAATGGTGATCATGTAACCCATCCTGACTTCATGTTGTATGACGCTCTTGATGTTGTTTTATACATGGACCCAATGTGCCTGGATGCGTTCCCAAAATTAGTTTGTTTTAAAAAACGTATTGAAGCTATCCCACAAATTGATAAGTACTTGAAATCCAGCAAGTATATAGCATGGCCTTTGCAGGGCTGGCAAGCCACGTTTGGTGGTGGCGACCATCCTCCAAAATCGGATCTGGTTCCGCGTGGATCCTGATCTAGATAGTTAAACCGCTGATCAGCCTCGACTGTGCCTTCTAGTTGCCAGCCATCTGTTGTTTGCCCCTCCCCCGTG |
| SQSTM1/P62 | TTCTGCTAGCGTTTAAACGGGCCCTCTAGACGCCACCATGGCGTCGCTCACCGTGAAGGCCTACCTTCTGGGCAAGGAGGACGCGGCGCGCGAGATTCGCCGCTTCAGCTTCTGCTGCAGCCCCGAGCCTGAGGCGGAAGCCGAGGCTGCGGCGGGTCCGGGACCCTGCGAGCGGCTGCTGAGCCGGGTGGCCGCCCTGTTCCCCGCGCTGCGGCCTGGCGGCTTCCAGGCGCACTACCGCGATGAGGACGGGGACTTGGTTGCCTTTTCCAGTGACGAGGAATTGACAATGGCCATGTCCTACGTGAAGGATGACATCTTCCGAATCTACATTAAAGAGAAAAAAGAGTGCCGGCGGGACCACCGCCCACCGTGTGCTCAGGAGGCGCCCCGCAACATGGTGCACCCCAATGTGATCTGCGATGGCTGCAATGGGCCTGTGGTAGGAACCCGCTACAAGTGCAGCGTCTGCCCAGACTACGACTTGTGTAGCGTCTGCGAGGGAAAGGGCTTGCACCGGGGGCACACCAAGCTCGCATTCCCCAGCCCCTTCGGGCACCTGTCTGAGGGCTTCTCGCACAGCCGCTGGCTCCGGAAGGTGAAACACGGACACTTCGGGTGGCCAGGATGGGAAATGGGTCCACCAGGAAACTGGAGCCCACGTCCTCCTCGTGCAGGGGAGGCCCGCCCTGGCCCCACGGCAGAATCAGCTTCTGGTCCATCGGAGGATCCGAGTGTGAATTTCCTGAAGAACGTTGGGGAGAGTGTGGCAGCTGCCCTTAGCCCTCTGGGCATTGAAGTTGATATCGATGTGGAGCACGGAGGGAAAAGAAGCCGCCTGACCCCCGTCTCTCCAGAGAGTTCCAGCACAGAGGAGAAGAGCAGCTCACAGCCAAGCAGCTGCTGCTCTGATCCCAGCAAGCCGGGTGGGAATGTTGAGGGCGCCACGCAGTCTCTGGCGGAGCAGATGAGAAAGATCGCCTTGGAGTCCGAGGGGCGCCCTGAGGAACAGATGGAGTCGGATAACTGTTCAGGAGGAGATGATGACTGGACCCATCTGTCTTCAAAAGAAGTGGACCCGTCTACAGGTGAACTCCAGTCCCTACAGATGCCAGAATCCGAAGGGCCAAGCTCTCTGGACCCCTCCCAGGAGGGACCCACAGGGCTGAAGGAAGCTGCCTTGTACCCACATCTCCCGCCAGAGGCTGACCCGCGGCTGATTGAGTCCCTCTCCCAGATGCTGTCCATGGGCTTCTCTGATGAAGGCGGCTGGCTCACCAGGCTCCTGCAGACCAAGAACTATGACATCGGAGCGGCTCTGGACACCATCCAGTATTCAAAGCATCCCCCGCCGTTGGGAGGTGGAGGATCATACCCTTATGATGTCCCAGACTATGCTTGATCTAGATAGTTAAACCGCTGATCAGCCTCG |

**Supplementary Table 3 | Primer sequences used for qPCR.**

| Symbol | Primer sequence (5′–3′) |
| --- | --- |
| NOTCH3-F | TGGCGACCTCACTTACGACT |
| NOTCH3-R | CACTGGCAGTTATAGGTGTTGAC |
| ACTA2-F | CTATGAGGGCTATGCCTTGCC |
| ACTA2-R | GCTCAGCAGTAGTAACGAAGGA |
| COL1A1-F | CATGGAGACTGGTGAGACCT |
| COL1A1-R | GCCATACTCGAACTGGAATC |
| COL1A2-F | GTTGCTGCTTGCAGTAACCTT |
| COL1A2-R | AGGGCCAAGTCCAACTCCTT |
| COL6A3-F | ATGAGGAAACATCGGCACTTG |
| COL6A3-R | GGGCATGAGTTGTAGGAAAGC |
| TAGLN-F | AGTGCAGTCCAAAATCGAGAAG |
| TAGLN-R | CTTGCTCAGAATCACGCCAT |
| MYO1B-F | CGGATGAAGCATACAGATCCC |
| MYO1B-R | CTGCCACATAGGACATGACAAG |
| GAPDH-F | ACCACAGTCCATGCCATCAC |
| GAPDH-R | TCCACCACCCTGTTGCTGTA |

**Supplementary Table 4 | Primer sequences used for ChIP–qPCR analysis of SMAD2 binding to the NOTCH3 promoter.**

| Symbol | Primer sequence (5′–3′) |
| --- | --- |
| Site1-F | GAGCGGAGATCATGCCACTG |
| Site1-R | CCCCCCACAATCTGCCAAGTA |
| Site2-F | CCTCCTGGGTTGAAGCGATT |
| Site2-R | AGAGGCAGAGGTGGATGGAT |
| Site3-F | ATCAGAGTCCTGGGGAAGCT |
| Site3-R | CTCTGGAGCCGGGACAAAAA |
